# Investigation of Intense Precipitation from Tropical Cyclones during the 21st Century by Dynamical Downscaling of CCSM4 RCP 4.5

**DOI:** 10.3390/ijerph16050687

**Published:** 2019-02-26

**Authors:** Mathieu Mure-Ravaud, M. Levent Kavvas, Alain Dib

**Affiliations:** Department of Civil and Environmental Engineering, University of California, Davis. 1 Shields Ave, Davis, CA 95616, USA; mlkavvas@ucdavis.edu (M.L.K.); aedib@ucdavis.edu (A.D.)

**Keywords:** dynamical downscaling, tropical cyclone, intense precipitation, general circulation model, climate change

## Abstract

In this article, a dynamical downscaling (DD) procedure is proposed to downscale tropical cyclones (TCs) from a general circulation model, with the goal of investigating inland intense precipitation from these storms in the future. This DD procedure is sequential as it is performed from the large scale to the small scale within a one-way nesting modeling framework with the Weather Research and Forecasting (WRF) model. Furthermore, it involves a two-step validation process to ensure that the model produces realistic TCs, both in terms of their general properties and in terms of their intense precipitation statistics. In addition, this procedure makes use of several algorithms such as for the detection and tracking of TCs, with the objective of automatizing the DD process as much as possible so that this approach could be used to downscale massively many climate projections with several sets of model options. The DD approach was applied to the Community Climate System Model (CCSM) version 4 using Representative Concentration Pathway (RCP) 4.5 during the period 2005–2100, and the resulting TCs and their intense precipitation were examined.

## 1. Introduction

General circulation models (GCMs) are useful tools to investigate future conditions on Earth. In particular, given the destructive potential of tropical cyclones (TCs), including their ability to produce intense precipitation (IP) that may in turn trigger disastrous floods, it is crucial to evaluate how these storms will evolve in a changing climate both in terms of their frequency and intensity. So far, mainly three approaches have been used to evaluate changes in TC properties due to climate change using GCMs.

The first approach is to use predictors for the development of TCs, which relate the observed frequencies of tropical cyclogenesis to large-scale environmental factors [1,2,3]. This approach has the advantage of being applicable at low resolution, of having a strong empirical foundation, and of being easy to apply to reanalysis or model datasets. However, several problems exist with current predictors, such as the overestimation of the importance given to sea surface temperature in some studies [4]. Moreover, this approach only accounts for TC location and frequency, and does not predict changes in the intensity or in the track of TCs. Finally, predictors are generally developed and calibrated to reproduce regional variability, and they might not be able to accurately respond to global changes [5].

The second approach consists of the direct use of GCM outputs to identify TCs. In order to be applicable, this approach requires a sufficient horizontal resolution to resolve TCs. However, due to the large computational cost associated with the run of a GCM, a compromise is generally looked for between the available computational power, the time available to perform the study, and the model resolution. In fact, in early studies, TCs were sometimes referred to as *hurricane-type vortices* [6] to emphasize the limitations of the models to reproduce actual TCs. One of the first attempts to simulate TCs was made by Broccoli and Manabe [7], who assessed the possibility of using a GCM to investigate the effects of greenhouse warming on the climatology of TCs using the Geophysical Fluid Dynamics Laboratory (GFDL) GCM with nine vertical layers and two horizontal resolutions: 7.5 × 4.5 and 3.75× 2.25 (longitude × latitude).

Although significant improvements have been made since then in the resolution of TCs in GCMs, the wide majority of the studies thus far [4,8] still acknowledge the limitations of GCMs to simulate the most intense TCs, such as Category 4 and 5 hurricanes on the Saffir–Simpson Hurricane Wind Scale [9]. Yet, a few recent studies reported promising results for the direct simulation of TCs by GCMs, proving that the next-generation GCMs may be able to resolve the full intensity spectrum of TCs. For example, Murakami et al. [10] investigated changes in TC activity in the future using a new version of the high-resolution 20- and 60-km-mesh Meteorological Research Institute (MRI) atmospheric GCM (MRI-AGCM version 3.2), and showed that the model was able to simulate very intense TCs (Category 4 and 5) reasonably well compared with observations, emphasizing that it was the first time that a GCM had been able to capture such intense TCs through a multidecadal simulation.

A third approach for the investigation of the change of TC properties in the future is *dynamical downscaling* (DD). DD allows to better resolve TCs since regional atmospheric models (RAMs) can be run with a finer resolution than GCMs. In the literature, DD has been used in several ways to study future TCs. Although the most intuitive DD approach would be to use a RAM to simulate to a finer resolution a storm present in the parent GCM, this approach has rarely been employed for TCs because, as mentioned previously, the TCs produced by a GCM may be poorly resolved and too weak. The most common alternative for the DD of TCs has been to use a large simulation outer domain, so that the RAM generates its own TCs. In particular, this approach has been used in a series of studies by Thomas R. Knutson and coworkers [11,12,13,14], in which case the modeling domain covered the tropical Atlantic, subtropical Atlantic, the Gulf of Mexico, and parts of western Africa.

However, Knutson et al. did not perform the direct DD of a GCM’s outputs. Indeed, they explained that GCMs have known biases which may distort the simulation of TCs. Instead, in these studies, they used a method usually referred to as the *pseudo-global warming dynamical downscaling* (PGW-DD) method, which is the most common approach for the DD of TCs under future conditions to date. The PGW-DD method consists of altering the present conditions, which may be obtained from an atmospheric reanalysis or from observation, by a time-invariant 3-dimensional climate change perturbation. Such a perturbation is obtained from a GCM or multi-GCM ensemble by subtracting the average conditions at the beginning of the considered period (e.g., early 21st century) from the average conditions at the end of this period (e.g., late 21st century). This approach has the advantage of reducing the model bias contained in the initial and boundary conditions (IBCs) from the GCM. On the other hand, the PGW-DD method makes strong assumptions: since the governing equations are nonlinear, adding perturbation fields due to climate change to the present-day atmospheric fields may result in imbalances between nonlinear terms, such as the advection terms [15].

Despite the concerns raised by the proponents of the PGW-DD method, a few studies reported on the direct DD of GCM outputs using a large simulation outer domain. For example, Done et al. [16] used the atmospheric component of the Nested Regional Climate Model (NRCM), a DD tool based on the Weather Research and Forecasting (WRF) model, to dynamically downscale the Community Climate System Model (CCSM) version 3 (CCSM3) with the A2 emissions scenario to investigate current and future TC activity in the North Atlantic, and to show the sensitivity and limitations of the DD approach by examining the effects of climate biases, resolution, and domain size. Several guidelines and precautions can be identified from this study for the purpose of performing the direct DD of a GCM’s outputs:

- The resolution in the simulation outer domain should be fine enough to resolve TCs;
- The outer domain should be large enough for several reasons. First, for the simulation of Atlantic TCs, the region of formation of African easterly waves should be included in the outer domain because these waves play an important role in the formation of TCs. Second, if the outer domain is too small, it will be too closely coupled with the driving GCM, and consequently unable to capture upscale interactions. This can be all the more problematic if the GCM is biased;
- The presence of bias in the driving GCM may prevent the RAM from generating its own TCs. In this case, it is necessary to remove the bias from the IBCs. In particular, in their study, Done et al. [16] found that the simulations driven directly by CCSM3 produced too strong large-scale flow at upper-levels over the tropical North Atlantic which was responsible for generating anomalously strong wind shear, thus preventing the genesis of TCs. They had to perform a bias correction of the driving GCM before running the model because no TCs were simulated with the biased IBCs. This bias correction was performed by expressing the 6-hourly CCSM3 data and the 6-hourly National Centers for Environmental Prediction (NCEP)/ National Center for Atmospheric Research (NCAR) Reanalysis Project (NNRP) data as the sum of a background state plus a perturbation. The background state was obtained by averaging over the 20-yr period from 1975 to 1994. Afterwards, the bias was removed by replacing the background state of CCSM3 by the background state of NNRP. They found that the wind shear over the tropical Atlantic was significantly improved when the NRCM was run with the bias-corrected IBCs, which allowed the genesis of TCs.

This article presents the results for the direct DD of CCSM version 4 (CCSM4) using Representative Concentration Pathway (RCP) 4.5 with the WRF model. More precisely, the article focuses on the simulation of IP from TCs during the 21st century. Section 2 presents the modeling framework. Section 3 discusses the detection and tracking algorithms (DTAs) used to identify TCs in the WRF model outputs. In Section 4, we ensure that the WRF model produced a realistic population of TCs by investigating several TC properties such as the maximum tangential surface wind speed (MTSWS). Section 5 deals with the DD to 15 km (intermediate domain) and 5 km (inner domain) horizontal resolutions. Section 6 presents the results in terms of the simulated IP from TCs during the 21st century. Finally, Section 7 presents some conclusions. It is noted that this article is accompanied by extensive supplementary material which will be referred to throughout the text, and which can be accessed from the journal’s website using the link provided after the article’s conclusions in Section 7.

## 2. Modeling Framework

The RAM used in this study was the Weather Research and Forecasting (WRF) model [17] version 3.7. The WRF model is a numerical weather prediction model used for operational and research applications to enhance the prediction and understanding of mesoscale weather and to accelerate the transfer of research advances into operations. The WRF model is a fully compressible and nonhydrostatic model. It is conservative for scalar variables and uses a terrain-following mass vertical coordinate in its vertical coordinate formulation.

The WRF model was selected among other available models for several reasons. First, it has been used extensively in recent studies for the simulation of TCs [18,19,20,21,22,23]. Besides, it is available for free and is easily downloadable and installable on a wide variety of Linux distributions. It also supports parallel computation with different memory architectures including distributed memory, shared memory, and distributed + shared memory. In addition, WRF has a large worldwide community of registered users, and there are workshops and tutorials on it each year at NCAR. Finally, the WRF model offers a wide range of model options including a relatively large number of parameterization schemes (PSs). It is noted that *parameterization* refers to the method of replacing processes that are too small-scale or complex to be physically represented in the model by a simplified process. Associated with these parameterizations are various parameters used in the simplified processes. A scheme refers to a given formulation of a parameterization (https://en.wikipedia.org/wiki/Parametrization_(atmospheric_modeling); last accessed on 18 February 2019).

For this study, the WRF model was run using 38 atmospheric model levels in the vertical direction with a pressure top of 50 mbar, a time step of 3 min, and the PSs of Table 1.

The GCM selected in this study was the Community Climate System Model version 4 (CCSM4). CCSM4 is a coupled climate model developed by NCAR to simulate the global climate system [24]. The model is composed of five separate components which simulate the Earth’s atmosphere, ocean, land, land-ice, and sea-ice simultaneously. It has a horizontal grid resolution of approximately 1.25 × 0.94 and 26 layers in the vertical direction.

GCMs, like CCSM4, are generally driven by Representative Concentration Pathways (RCPs), which are greenhouse gas concentration trajectories for the future [25,26]. These RCPs describe a total of four possible climate futures: one mitigation scenario (RCP 2.6), two intermediate stabilization scenarios (RCP 4.5 and RCP 6.0), and one very high baseline emission scenario (RCP 8.5). The numerical values of the RCP scenarios represent the radiative forcing values of the year 2100 relative to pre-industrial levels, measured in watts per square meter (W m${}^{-2}$) [26,27]. In this study, one intermediate stabilization scenario, RCP 4.5, was used, corresponding to an increase of the radiative forcing relative to pre-industrial levels of 4.5 W m${}^{-2}$ by 2100. CCSM4 RCP 4.5 (denoted hereafter simply as CCSM4) data are available for the period 2005–2100. These data may be downloaded from the Climate Data Gateway at NCAR (https://www.earthsystemgrid.org/; last accessed on 18 February 2019) or from the archives of the German Climate Computing Center (DKRZ; https://cera-www.dkrz.de/WDCC/ui/cerasearch/; last accessed on 18 February 2019).

**Figure 1:**
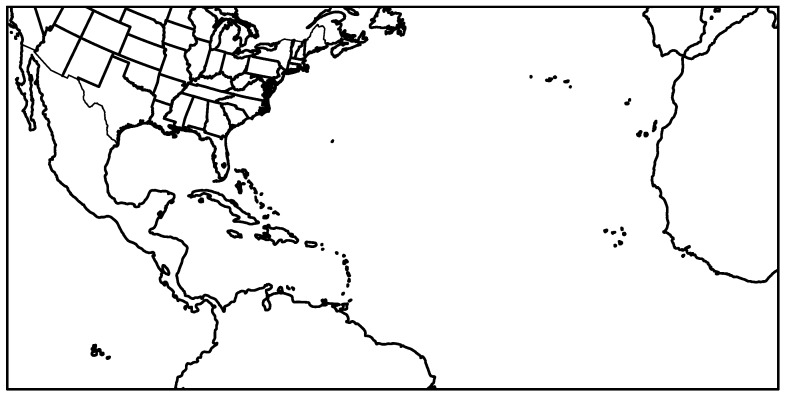
Simulation domain used for the first DD step at 45 km horizontal resolution. It is composed of 240 × 120 grid points.

Examination of CCSM4 data revealed that the GCM failed to produce a population of well-defined TCs. Indeed, TCs are too few and too weak, which is not surprising given the relatively coarse resolution of the model. This problem was tackled by using the DD alternative discussed in Section 1: a large simulation outer domain was used (Figure 1), which encompasses the eastern Atlantic Ocean and the western coast of Africa (corresponding to the region of formation of African easterly waves), so that the WRF model will hopefully produce its own TCs.

More precisely, the following line of actions was employed for the DD of CCSM4 with WRF:

1. Perform the DD of CCSM4 with the WRF model at 45 km horizontal resolution with the domain shown in Figure 1 for all hurricane seasons during the period 2005–2100. One hurricane season was defined as the time period between the beginning of July and the end of November;
2. Examine the WRF model outputs from the previous step to assess whether the model generated a sufficiently large number of TCs, including a sufficiently large number of landfalling TCs for the study of IP over the eastern and southern U.S. in the future. If so, one can go to the next step. It is noted that since the focus of this study is on IP rather than TC climatology (i.e., TC frequency, geographical distribution, seasonal variability, etc.), it is less problematic if the number of TCs simulated by the WRF model is less or more than it should be. There should be, however, substantially more TCs in the WRF model outputs than in CCSM4, since the scarcity of TCs in CCSM4 was the main reason for adopting this DD alternative. If the model fails to produce enough TCs, additional work is required. For example, following the discussion in Section 1, one may try to remove the bias from CCSM4 and run the WRF model with the unbiased IBCs;
3. Examine the TCs in the WRF model outputs to assess whether their properties (e.g., MTSWS) are realistic. For example, did the model generate a population of TCs that is overall too strong, or too weak? If not, one can go to the next step. Otherwise, additional work is required, which again may involve trying to remove the bias from CCSM4 and run the WRF model with the unbiased IBCs;
4. Perform DD to 15 km horizontal resolution of the TCs identified in Step 2;
5. Perform DD to 5 km horizontal resolution of the results from Step 4;
6. Validate the WRF model performance in simulating IP from TCs during the historical period (corresponding to 2005–2017 in this study). This step is necessary if one wants to confidently use the WRF model previsions of future IP from TCs.

**Figure 2:**
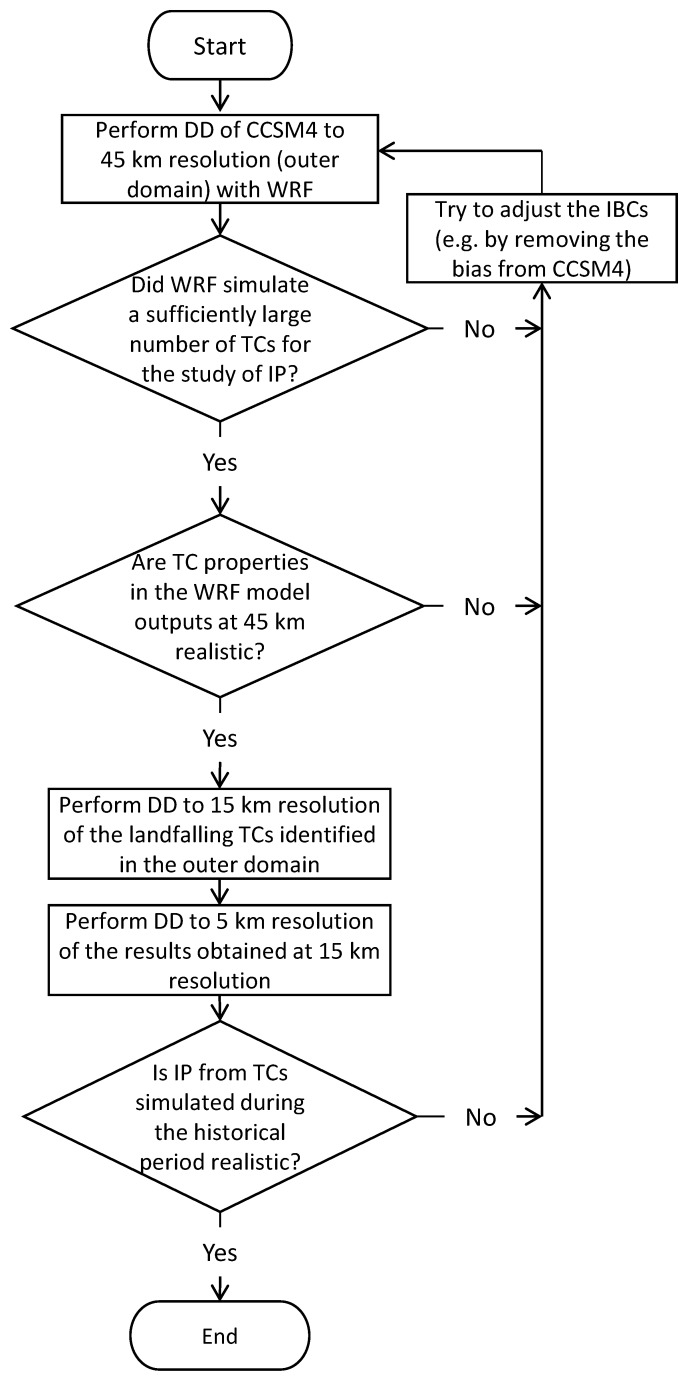
Flowchart of the steps for the DD of CCSM4 with WRF. IBC: initial and boundary condition, IP: intense precipitation, TC: tropical cyclone.

The flowchart of these steps is given in Figure 2. It is noted that, in this line of actions, the DD of future TCs is performed sequentially, from the large scale simulated at 45 km resolution to the small scale simulated at 5 km resolution. Technically, this implies that one-way nesting must be used. *One-way nesting* means that the nested domains are run separately and sequentially starting with the outer domain, which differs from a *two-way nesting* modeling framework for which the nested domains are run simultaneously and communicate with each other.

## 3. Detection and Tracking of Tropical Cyclones

TCs in the WRF model outputs at 45 km resolution were identified using detection and tracking algorithms (DTAs). Indeed, although TCs could have been identified manually by visual examination of the model outputs, using for example the plots of the sea-level pressure (SLP) field (which exhibits a local minimum at the location of a TC) or of the meridional surface wind speed (SWS) field (which exhibits a dipole at the location of a TC), using such algorithms has the advantage of significantly limiting the time of postprocessing, so that the DD approach presented in this article may be applied to many climate projections and/or combinations of the model options. In this study, DD was performed for only one climate projection (CCSM4 RCP 4.5) and one combination of the PSs (Table 1). Yet, several studies [12] have found that DD results in terms of TC activity during the 21st century may vary dramatically when the GCM used for the IBCs is modified, highlighting the importance of performing multiple simulations of future conditions before drawing any solid conclusions.

The detection algorithm was adapted from Bengtsson et al. [6], who used the ECHAM3 atmospheric model (ECHAM is an atmospheric GCM developed at the Max Planck Institute for Meteorology; http://www.mpimet.mpg.de/en/science/models/echam.html; last accessed on 18 February 2019) to investigate the influence of greenhouse warming on the climatology of TCs. In this algorithm, TCs are identified by using several physical and dynamical criteria. More specifically, several thresholds are used for different quantities such as the relative vorticity at 850 hPa. In the present study, two separate sets of criteria were adopted: a first set called *high-threshold* set which allowed the detection of intense TCs, and a second, less stringent set called *low-threshold* set which was used for the reconstruction of their tracks. Model grid points passing the criteria were then gathered into TCs based on a distance threshold. Next, TC tracks were reconstructed by matching TCs at a given time step with TCs at the adjacent time steps using a threshold on the TC speed. It is noted that, for this study, the objective of these algorithms was not to necessarily detect all TCs, but a sufficient number of these storms to allow the investigation of their IP properties during the 21st century. More precisely, the DTAs were designed to be loose enough to detect as many TCs as possible but conservative enough to limit unintended postprocessing work to remove manually spurious storms from the TC population. A more extensive description of these algorithms is provided in the supplementary material *Suppl.Mat._Detection_and_Tracking_algorithms.pdf*.

The DTAs were first applied to the Climate Forecast System Reanalysis (CFSR) for 2004–2017 in order to assess their performance. Table 2 gives the number of TCs for three categories: storms of tropical storm (TS) strength or more, storms of hurricane strength or more, and major hurricanes (i.e., Category 3, 4 and 5 hurricanes). It shows that the DTAs’ performance strongly depends on TC intensity: almost all major hurricanes were identified by the algorithms versus less than half of the TSs. As far as the simulation of IP is concerned, this behavior is a limitation because the largest rain-producing TCs are not always the most intense storms based on the wind speed. For example, TS Fay (2008) and TS Lee (2011) spawned torrential precipitation in the U.S. However, since the most intense TCs according to the Saffir–Simpson Hurricane Wind Scale generally produce IP, identifying such TCs should allow a large fraction of the population of the largest rain-producing TCs to be reconstructed while missing a few of them associated with a weaker physical and thermodynamical signature (e.g., lower wind speeds and lower central pressure deficits (CPDs)). Besides, the plots of the reconstructed and observed TC tracks for Category 4 and 5 hurricanes during the period 2004–2017 are provided in the supplementary material *Suppl.Mat._Validation_Detection&Tracking.pdf*. This document shows that the DTAs performed well in reconstructing the tracks of these intense hurricanes.

The DTAs were then applied to the WRF model outputs for the DD of CCSM4 to 45 km resolution. A total of 49 TCs were detected during the period 2005–2017, including 11 landfalling TCs. This is less than the number of TCs detected in CFSR (76 TCs were detected in CFSR during the period 2005–2017), but this still offers a large enough population of TCs to work with. In particular, there was, on average, almost one landfalling TC per year during the period 2005–2017 in the WRF model outputs. Besides, 393 TCs were detected during the period 2005–2100 including 64 landfalling TCs. The supplementary material *Suppl.Mat._Application_DTAs_21st_century.pdf* discusses the evolution of the number of landfalling and non-landfalling TCs in the future.

## 4. Calculation of the Properties of Tropical Cyclones

In Section 3, it was shown that the WRF model succeeded in producing a sufficiently large population of TCs (at 45 km resolution) to work with for the purpose of this study, and that the proposed DTAs allowed a large fraction of these TCs to be identified despite the rather conservative choices made in the design of these algorithms. In this context, what is meant by *sufficiently large* is that the number of landfalling TCs identified in the model outputs was large enough to compute statistics for IP from this ensemble of storms. More precisely, there should be a sufficiently large number of realizations of an inland precipitation depth (PD) field during the historical period (2005–2017 for this study) to assess whether the model is suitable for the simulation of IP from TCs. On the other hand, the number of landfalling TCs during the 21st century should be sufficiently large to allow computing such IP statistics for the future conditions. These aspects are further discussed in Section 6 where four IP metrics are introduced to quantify the intensity of precipitation from TCs. Thus, the second step of the line of actions proposed in Section 2 is checked out. The next step is to assess whether the population of TCs identified so far is realistic in terms of its TC properties. The TC properties investigated in this study are the central pressure deficit (CPD), maximum tangential surface wind speed (MTSWS), radius of MTSWS (RMTSWS), TC characteristic horizontal length (TCCHL), and TC depth.

### 4.1. Calculation of the CPD and TCCHL

The CPD and TCCHL were first computed for each TC and each time step during a TC’s lifetime by analyzing the radial profile of the SLP. Although the SLP field generally already exhibits a clear local minimum at the TC location, we went one step further and worked instead with a SLP perturbation field. This perturbation field was computed by subtracting from the original SLP field a mean SLP field obtained by averaging the SLP over all time steps during the hurricane seasons of 2005–2100. The advantage of this exercise is that the local depression at the TC location in the SLP perturbation field is even more well-defined than in the original SLP field.

**Figure 3:**
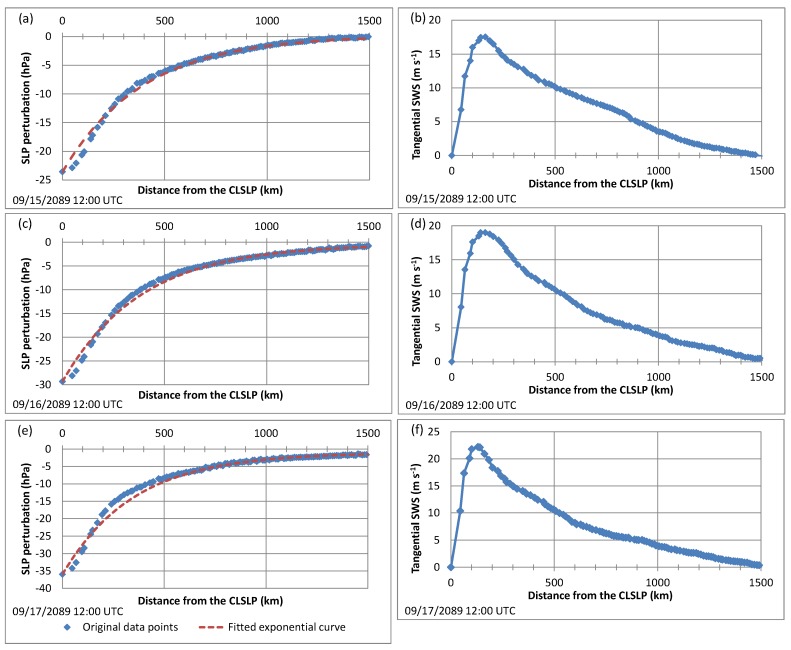
Graphs of the azimuthally-averaged sea level pressure (SLP) perturbation as a function of the distance from the center of low SLP (CLSLP) in a TC in September 2089 for three dates: (**a**) 09/15 12:00 UTC, (**c**) 09/16 12:00 UTC, and (**e**) 09/17 12:00 UTC. The red dashed line corresponds to the fitted exponential curve used to estimate the TC characteristic horizontal length (TCCHL); Graphs of the azimuthally-averaged tangential surface wind speed (SWS) as a function of the distance from the CLSLP in a TC in September 2089 for three dates: (**b**) 09/15 12:00 UTC, (**d**) 09/16 12:00 UTC, and (**f**) 09/17 12:00 UTC.

Secondly, all the grid points within a distance of 2000 km from the TC’s center of low SLP (CLSLP) were identified and used to compute the radial profile of the SLP perturbation field by averaging this field azimuthally. An example is provided in Figure 3. More precisely, the blue curves in Figure 3a,c,e provide the radial profiles of the SLP perturbation field for three dates in a TC in September 2089. The CPD was taken as the difference between the SLP perturbation far from the CLSLP (usually close to 0) and the SLP perturbation at the CLSLP. On the other hand, it was observed that the radial profiles of SLP perturbation are roughly exponential, so exponential curves (red curves in Figure 3a,c,e) were fitted to these radial profiles in order to determine the TCCHL. More precisely, the TCCHL was determined by fitting the following function to the radial profile of SLP perturbation:

(1) $$\Delta P\left(r\right)=\Delta P\left(+\infty \right)-\mathrm{CPD}\times \exp\left(-\frac{r}{\mathrm{TCCHL}}\right)$$

where *r* is the distance from the TC CLSLP and ΔP is the SLP perturbation. Note that ΔP(+∞) and CPD are known at this stage, so that the TCCHL can be estimated using the data. The fitting was performed by calculating the characteristic length parameter for each data point $r_{i}$:

(2) $${\mathrm{TCCHL}}_{i}=\frac{r_{i}}{\ln\left(\frac{\mathrm{CPD}}{\Delta P\left(+\infty \right)-\Delta P\left(r_{i}\right)}\right)}$$

and by taking the mean of the ${\mathrm{TCCHL}}_{i}$.

### 4.2. Calculation of the MTSWS and RMTSWS

Similar to the case of the SLP field, a mean SWS field was computed by taking the average over all time steps during the hurricane seasons of 2005–2100. This mean SWS field was then subtracted from the SWS field at the considered time step in order to obtain an SWS perturbation field. The advantage of working with this perturbation field is that it is generally free from strong background currents such as the trade winds, so that the circulation around the CLSLP is more axisymmetric than in the original field. In fact, we went one step further by calculating the mean SWS perturbation within a radius of TCCHL from the CLSLP and subtracted this mean value from the SWS perturbation field, so that the resulting field is even closer to being axisymmetric. These efforts were done in an attempt to separate the TC circulation from the background field to improve the estimation of the MTSWS and RMTSWS.

Next, all the grid points located within a distance of 2×TCCHL from the CLSLP were identified and used to compute the radial profile of the tangential SWS by averaging the tangential SWS azimuthally. Examples of such radial profiles are provided in Figure 3b,d,f for three dates in a TC in September 2089. Eventually, the MTSWS was taken as the maximum value of the tangential SWS radial profile whereas the RMTSWS was taken as the distance from the CLSLP for which this maximum value is attained.

### 4.3. Calculation of the TC Depth

Finally, the TC depth was estimated using the vertical profile of the vertical vorticity above the CLSLP. It was defined as the height for which the vertical vorticity drops below a given threshold taken as 5% of the surface vertical vorticity.

### 4.4. Comparison of TC Properties between CFSR and the WRF Model Outputs during the Period 2005–2017

In order to assess the adequacy of the TCs produced by the WRF model, the aforementioned TC properties were calculated for each TC identified in the WRF model outputs at 45 km resolution and in CFSR, and their mean values were compared (Table 3).

For the comparison of TC properties to be legitimate, the spatial resolution of the data should be the same, or at least should be close. The horizontal resolution of the WRF model outputs is 45 km whereas it is 0.5 for CFSR. 0.5 of latitude is approximately equal to 55.5 km whereas the distance in kilometers corresponding to 0.5 of longitude varies with latitude. For example, at the equator, 0.5 of longitude is approximately equal to 55.6 km while at 40N it is about 42.5 km. Besides, CFSR uses 37 atmospheric vertical levels whereas the WRF model was run with 38 levels. As a result, in the region of investigation for this study, the horizontal and vertical resolutions of the WRF model outputs and of CFSR are similar, although resolution is slightly coarser for CFSR. It is consequently legitimate to compare the second and third columns of Table 3.

Table 3 shows that TCs in the WRF model outputs are slightly more intense than in CFSR. Indeed, they have, on average, a larger CPD and a larger MTSWS. On the other hand, the RMTSWS, TCCHL, and TC depth are, on average, smaller in the WRF model outputs than in CFSR, so that TCs are smaller in size in the WRF model outputs. Should it be concluded that the WRF model failed to produce realistic TCs? Not necessarily. Indeed, these results may be explained by the slightly coarser resolution of CFSR compared to the WRF model outputs, which causes TCs to be slightly weaker and larger in CFSR. This tendency of a coarser model to produce larger and weaker TCs has, in fact, been reported in several studies which looked at the impacts of different resolutions on simulated TCs [20,28,29,30,31,32]. All these studies, among others, reported higher wind speeds, lower radii of maximum wind speeds, lower minimum SLP, and in general a higher intensity of TCs that were simulated at finer resolutions when compared to TCs simulated at a coarser resolution.

Given this potential explanation in terms of the model resolution, and the fact that the difference between the two datasets is relatively small, it was decided that the population of TCs produced by the WRF model is sufficiently realistic for the purpose of this study, which checks out the third step of the line of actions proposed in Section 2. Besides, the evolution of TC properties during the 21st century is discussed in the supplementary material *Suppl.Mat._TC_properties_21st_century.pdf*.

## 5. Dynamical Downscaling to 15 km and 5 km Resolutions

Section 3 and Section 4 showed that, within the modeling framework proposed in this study, the WRF model succeeded in simulating a sufficiently large and realistic population of TCs, so that we can move to the next steps in the line of actions of Section 2. These steps involve the DD to 15 km and 5 km resolutions of the TCs identified previously in the WRF model outputs at 45 km resolution. More precisely, since this study focuses on inland IP from TCs, only landfalling TCs were further downscaled to 15 and 5 km resolutions.

One of the issues in the choice of the intermediate and inner domains for the simulation of IP from TCs in the eastern and southern U.S. is the diversity of TC tracks and consequently of landfall locations. This aspect may be tackled in two ways: either by customizing the nested domains to each storm so that they fit the location and size of the PD field, or by taking large nested domains encompassing the whole eastern and southern U.S. The problem with the first approach is that it may be time consuming to set up the nested domains manually for every storm. On the other hand, the problem with the second approach is that the time of computation increases drastically with the model resolution, so that it may not be feasible to perform the DD of such a large region to a fine resolution.

A compromise was chosen in this study. First, it was found that the computational effort associated with the DD with a large domain of each TC in the WRF model outputs at 45 km resolution to 15 km resolution remained reasonable, so that such a large intermediate domain was used for the first downscaling step to 15 km resolution. Besides, for a given TC, the intermediate-domain simulation start date was taken as the date for which this TC’s MTSWS was the largest as the storm was moving over the Atlantic Ocean.

Secondly, as far as the inner domain is concerned, it was chosen to construct this domain case by case, according to the location and size of each TC’s PD field in the intermediate domain. Algorithms were developed for this purpose. Indeed, setting up the inner domain manually for each storm would be very time consuming, which goes against the aforementioned objective in this work of automatizing the DD procedure as much as possible. The algorithms for the determination of the inner-domain simulation start and end dates and for the construction of the inner domains based on the PD fields in the intermediate domain are detailed in the supplementary material *Suppl.Mat._Creation_Inner_Domain.pdf*.

In a nutshell, in these algorithms, the inner-domain simulation start date is taken one day before the time of landfall whereas the inner-domain simulation end date is taken as the time for which a TC stops producing IP over the U.S. The inner domain is then constructed based on the PD field in the intermediate domain accumulated between the inner-domain simulation start and end dates. The simulation start and end dates in the intermediate and inner domains for the 64 landfalling TCs during the 21st century are given in Appendix A. It is noted that the aforementioned efforts to reduce the size of the inner domains and the duration of the simulations in the inner domains by using algorithms to automatically detect where and when a given TC hit the country resulted in a drastic decrease in the computational effort associated with the WRF model simulations at the finest resolution of 5 km. The inner-domain PD fields of the 64 landfalling TCs are shown in Appendix B.

## 6. Intense Precipitation from Tropical Cyclones during the 21st Century

In the previous section, the DD of North Atlantic TCs during the 21st century was performed to 15 and 5 km resolutions to simulate to a fine resolution the PD fields produced by these TCs in the eastern and southern U.S. However, in order to confidently and legitimately use the WRF model’s previsions of IP from TCs in the future, one must first verify that IP simulated during the historical period of 2005–2017 is realistic. This task corresponds to the last step of the line of actions proposed in Section 2.

### 6.1. What Is Intense Precipitation?

First and foremost, what is *intense precipitation* (IP)? This notion is, in fact, very subjective. In the literature, many definitions have been used. For example, Kunkel et al. [33] defined an extreme rainfall event as an event exceeding a 5-yr return period, which corresponds to a threshold ranging from less than 25 mm in some regions of the interior west to more than 200 mm along the Gulf Coast. In Schumacher and Johnson [34], an event was considered extreme if one or more gauges reported a 24-h PD greater than the 50-yr recurrence interval PD for that location as determined by Hershfield [35], which corresponds to a threshold ranging from less than 102 mm in some regions of the interior west to more than 330 mm along the Gulf Coast. These differences between studies are explained by the choice of different return periods, accumulation intervals, datasets, etc. In this study, we defined four metrics to quantify the intensity of a PD field.

**• IP Metric 1:** surface area of the PD field above a given threshold 

In Stevenson and Schumacher [36], an IP event was defined as an event for which at least one grid point in the Stage IV precipitation dataset showed a PD larger than a predefined threshold. Stage IV is a mosaic of regional multi-sensor precipitation analysis produced by National Weather Service (NWS) River Forecast Centers since December 2001 [37,38]. It has been used extensively in recent studies [36,39,40]. Stage IV combines rain gauge data and radar-estimated rainfall. It is provided at 4 km horizontal resolution, and three time resolutions: 1 h, 6 h, and 24 h. Four thresholds were considered in Stevenson and Schumacher [36]: 50-yr return period/6-h accumulated PD, 100-yr return period/6-h accumulated PD, 50-yr return period/24-h accumulated PD, and 100-yr return period/24-h accumulated PD. The threshold values were obtained from the U.S. Weather Bureau Technical Paper No. 40 [41]. The 10 events with the most IP were then identified according to the number of grid points in Stage IV above the 100-yr, 24-h threshold.

It is noted that this quantification (number of grid points above a threshold) depends on the resolution of the precipitation dataset. Indeed, the finer the resolution is, the larger the number of grid points above the threshold is. As a result, in this study, IP Metric 1 was instead taken as the **surface area** of the region where the PD field exceeds the PD threshold field. Still, IP Metric 1 remains equivalent to the metric used in Stevenson and Schumacher [36] since it is obtained by multiplying the number of grid points above the threshold by the surface area of one grid cell, but it has the advantage of being independent of the resolution of the precipitation dataset. Mathematically, IP Metric 1 can be written as:

(3) $$\mathrm{IP}\;\mathrm{Metric}\;1=\max_{t}\left(\;\;\;\underset{\mathrm{Domain}}{\;\int \int \;}𝟙\left\{\mathrm{PD}\left(\bar{x},t\right)\geq \mathrm{threshold}\left(\bar{x}\right)\right\}\;d\bar{x}\right)$$

where Domain refers to the region under investigation (e.g., simulation inner domain for the WRF model outputs), $𝟙\left\{•\right\}$ is the indicator function which is equal to 1 if the condition between brackets is met and equal to 0 otherwise, $\bar{x}$ gives the spatial location, and *t* is time. The max operator suggests that the maximum value over the duration of the storm should be retained. For example, suppose that a storm lasts for 48 h, that one is interested in the 24-h PD, and that the precipitation data is provided with a 1-h time increment. In this case, there will be 25 realizations of a 24-h PD field within the 48-h time interval, and the maximum of these 25 realizations should be retained in the construction of the metric. In other words, IP Metric 1 gives the surface area of the region where the PD field is larger than the PD threshold field at the time when the storm is the most intense.

**• IP Metric 2:** weighted surface area of the PD field 

IP Metric 1 contains information regarding whether a PD field exceeded a given threshold. However, it cannot tell how much larger than the threshold the PD field was. For example, let us consider two storms: the first storm produced a PD field with 1000 km^2^ slightly above the PD threshold field whereas the second storm produced a PD field with 200 km^2^ where the PD threshold field was greatly exceeded. According to IP Metric 1, the first storm is much more intense than the second. However, is such a conclusion legitimate?

IP Metric 2 is proposed to tackle the emphasized limitation of IP Metric 1. In this case, the PD field is normalized using the PD threshold field, and the resulting normalized PD field is then integrated over the domain of investigation. Mathematically, IP Metric 2 is given by:

(4) $$\mathrm{IP}\;\mathrm{Metric}\;2=\max_{t}\left(\;\;\;\underset{\mathrm{Domain}}{\;\int \int \;}\frac{\mathrm{PD}(\bar{x},t)}{\mathrm{threshold}(\bar{x})}\;d\bar{x}\right)$$

IP Metric 2 is clearly a generalization of IP Metric 1.

**• IP Metric 3:** maximum PD over a size-predefined area 

IP Metrics 1 and 2 provide information on the intensity of precipitation at the spatial scale of the storm, which may be a very large region encompassing several states. As a result, they cannot quantify IP at a predefined scale. For example, in the context of Probable Maximum Precipitation (PMP; the greatest PD for a given duration meteorologically possible for a design watershed or a given storm area at a particular location at a particular time of the year, with no allowance made for long-term climatic trends [42]) estimation, the target area is fixed, and it may be either much smaller or much larger than an individual storm. IP Metrics 3 and 4 are introduced to tackle this issue.

IP Metric 3 is given by the maximum PD that a TC spawned over an area of a given size in a given time interval. For example, this may be the maximum PD falling over an area of 2400 km^2^ in 72 h. More generally, instead of one value, one may consider the depth–duration curve for this specified area by looking at a range of time intervals. In fact, one may even go one step further and compute the depth–area–duration curves, in which case not just one, but a range of spatial scales are accounted for. However, the computation of the depth–area–duration curves can be difficult and computationally intensive.

**• IP Metric 4:** maximum normalized PD over a size-predefined area 

IP Metric 4 is constructed in the same way as IP Metric 3, but it uses the normalized PD field instead of the original (dimensional) PD field. As for IP Metric 2, the normalized PD field is obtained by dividing the PD field by the PD threshold field. Using the previous example, if IP Metric 4 is equal to 3.0, this means that, within the domain of investigation (e.g., the simulation inner domain), the largest PD that fell over an area of 2400 km^2^ in 72 h was, on average, three times larger than the threshold.

### 6.2. Validation of the WRF Model Performance in Simulating IP from TCs during the Period 2005–2017

In order to validate the WRF model performance in simulating IP from TCs, the four IP metrics were calculated during the period 2005–2017 for all landfalling TCs in the WRF model outputs, and for all historical landfalling TCs. Stage IV was used for the historical TCs in the calculation of the IP metrics. Historical landfalling TCs were determined by visual examination of TC tracks provided by the free online encyclopedia *Wikipedia* by checking if these tracks passed over the conterminous U.S. (Wikipedia provides a detailed description of every hurricane season; for example, for 2004, see https://en.wikipedia.org/wiki/2004_Atlantic_hurricane_season; last accessed on 18 February 2019), whereas the dates and times of landfall and termination were obtained from the Tropical Cyclones Reports of the National Hurricane Center. The list of historical landfalling TCs during the period 2005–2017 is given in the supplementary material *Suppl.Mat._Historical_Landfalling_TCs.pdf*.

For each of the historical landfalling TCs, the region affected by the TC’s precipitation was then determined by using the algorithms for the construction of the simulation inner domain discussed in Section 5. This step does not aim at creating a simulation inner domain per se since historical TCs are not to be dynamically downscaled, but rather to limit the size of the region used for the calculation of the IP metrics. Indeed, since Stage IV encompasses most of the U.S., using the entire domain of the Stage IV data for the calculation of the IP metrics may lead to the inclusion of other storm systems which would result in misestimating the metrics.

**Figure 4:**
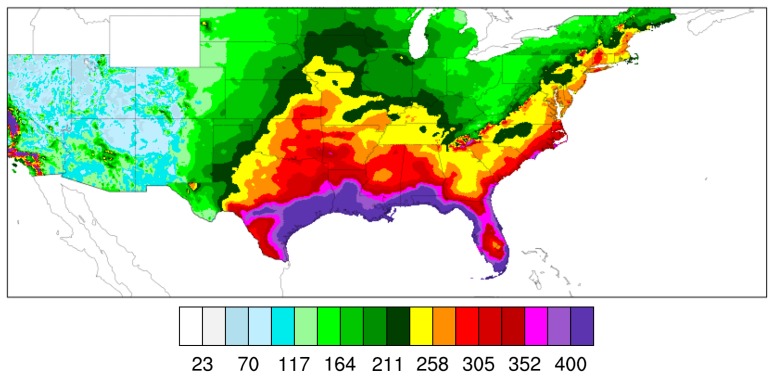
National Oceanic and Atmospheric Administration (NOAA) Atlas 14 100-yr, 72-h precipitation depth (mm) threshold field (for all U.S. states except the northwestern states).

The IP metrics were calculated using the NOAA Atlas 14 100-yr, 72-h PD threshold field (Figure 4) obtained from the Precipitation Frequency Data Server (PFDS) of the National Weather Service (NWS)’s Hydrometeorological Design Studies Center (https://hdsc.nws.noaa.gov/hdsc/pfds; last accessed on 18 February 2019). A 72-h period was considered instead of the 24-h period used in Stevenson and Schumacher [36] because a 72-h period seemed more suited to the case of TCs since these systems usually last for several days.

A simplified approach was used to compute IP Metrics 3 and 4. When looking for the maximum PD (IP Metric 3) or the maximum normalized PD (IP Metric 4) that fell over an area of a given size, all geometries for this area should be ideally considered. However, such an approach would be computationally very intensive. As a result, a square region was considered for the calculation of IP Metrics 3 and 4. More precisely, considering an area of size S km^2^, IP Metrics 3 and 4 were calculated by using a square of side $\sqrt{S}$ and by moving this square in the region affected by the TC PD field (corresponding to the simulation inner domain in the case of the WRF model outputs) to find the maximum.

IP Metrics 3 and 4 were calculated using a spatial scale of 2400 km^2^. The reason for the choice of a region size of 2400 km^2^ is the following. The material presented in this article actually originates from the third chapter of a Ph.D. thesis [43] entitled *On the maximization of precipitation from tropical cyclones in the context of climate change*. In Section 2 of this thesis, a physically based storm transposition method for TCs is presented and applied to four hurricanes taking the drainage basin of the city of Asheville, NC as the target area. In Section 3, this storm transposition method is also applied to the two most intense future TCs (which is not presented in this article) with the same target area. The drainage basin of the city of Asheville has a surface area of 2400 km^2^, which explains why IP Metrics 3 and 4 are calculated based on a surface area of 2400 km^2^ in this article.

**Figure 5:**
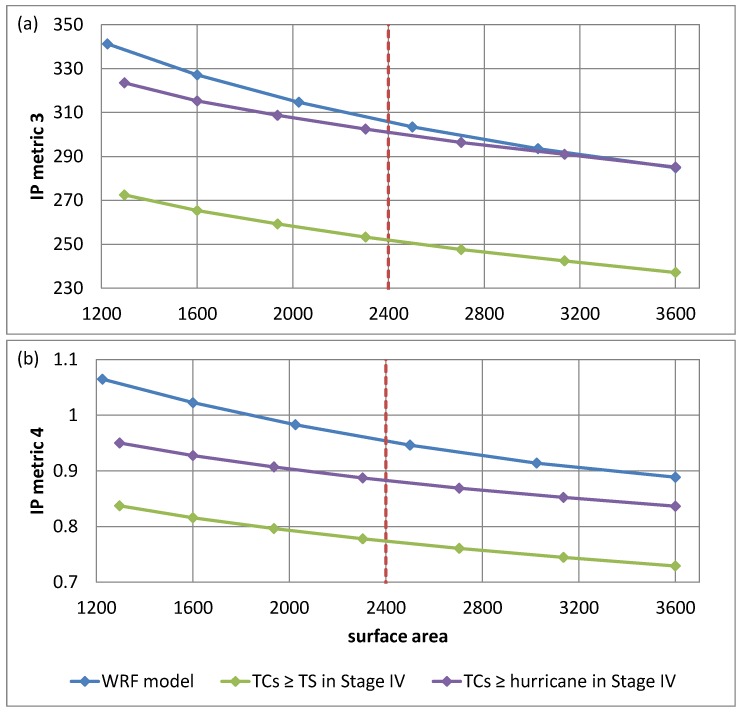
WRF model validation results for the simulation of IP during the period 2005–2017 measured by (**a**) IP Metric 3 (mm) and (**b**) IP Metric 4 for a range of surface areas (km^2^). These are average metrics calculated by averaging the metrics of all landfalling TCs during the period 2005–2017. The blue curve gives the metrics for the WRF model outputs whereas the other two curves were obtained by computing the metrics using the Stage IV precipitation dataset in the case where all landfalling TCs with strength ≥ TS strength are considered (green curve), and in the case where all landfalling TCs with strength ≥ hurricane strength are considered (purple curve). The dotted red line indicates a surface area of 2400 km^2^.

More precisely, several surface areas around 2400 km^2^ were considered for the purpose of the WRF model validation. In the case of the WRF model outputs in the inner domains (5 km resolution), squares of sides 7 grid cells, 8 grid cells, 9 grid cells, 10 grid cells, 11 grid cells and 12 grid cells were used which correspond to surface areas of 1225 km^2^, 1600 km^2^, 2025 km^2^, 2500 km^2^, 3025 km^2^, and 3600 km^2^, respectively. In the case of Stage IV having a horizontal resolution of 4 km, squares of sides 9 grid cells, 10 grid cells, 11 grid cells, 12 grid cells, 13 grid cells, 14 grid cells and 15 grid cells were used which correspond to surface areas of 1296 km^2^, 1600 km^2^, 1936 km^2^, 2304 km^2^, 2704 km^2^, 3136 km^2^, and 3600 km^2^, respectively. Validation results are given in Table 4 for IP Metrics 1 and 2, and in Figure 5 for IP Metrics 3 and 4.

Table 4 shows that results in terms of the mean and standard deviation of IP Metric 1 are satisfactory. Indeed, for both the mean and standard deviation, IP Metric 1 for the simulated precipitation is larger than IP Metric 1 for the observed precipitation (from Stage IV) when this observed precipitation is taken for all TCs of strength at least equal to TS strength. However, IP Metric 1 for the simulated precipitation is smaller than IP Metric 1 for the observed precipitation when the observed precipitation is taken for all TCs of strength at least equal to hurricane strength. This result is not surprising because the TC DTAs used to identify TCs in the WRF model outputs at 45 km resolution perform best with the most intense TCs, and only manage to capture a small fraction of the TSs (see Table 2).

As far as IP Metric 2 is concerned, results are less satisfactory. Indeed, the mean and standard deviation of IP Metric 2 for the simulated precipitation are smaller than the mean and standard deviation of IP Metric 2 for the observed precipitation in both the aforementioned cases (i.e., TCs ≥ TS and TCs ≥ hurricane). This suggests that the WRF model tended to produce too compact PD fields in the DD of CCSM4 when using the PSs of Table 1.

Besides, Figure 5 provides the mean of IP Metrics 3 and 4 for a range of region sizes and for the three cases discussed previously: (1) IP in the WRF model outputs (blue curve), (2) IP in Stage IV for TCs ≥ TS (green curve) and (3) IP in Stage IV for TCs ≥ hurricane (purple curve). Note that Figure 5a actually gives the 72-h depth–area curves whereas Figure 5b provides a normalized version of these curves. Results in terms of the mean of IP Metrics 3 and 4 are satisfactory, although IP at these scales was slightly overestimated by the WRF model since IP Metrics 3 and 4 for the simulated precipitation were on average larger than IP Metrics 3 and 4 for the observed precipitation in both the cases TCs ≥ TS and TCs ≥ hurricane.

Thus, the WRF model gave overall satisfactory performance in simulating IP during the historical period 2005–2017, despite a tendency to produce slightly too compact and too locally intense PD fields compared to the PD fields in Stage IV. It is noted that, in practice, to save time, DD should be first performed for the historical period only, and the future years be downscaled only after validation of the model performance in simulating both TC properties (as in Section 5) and IP from TCs during the historical period.

### 6.3. Evolution of IP from TCs during the 21st Century

In this subsection, the IP metrics are calculated for all landfalling TCs in the WRF model outputs during the 21st century using the 100-yr, 72-h PD threshold field of Figure 4. IP Metrics 3 and 4 were calculated only for a region of 2500 km^2^, which corresponds to a square having 10 grid cells per side for the searching region since the resolution of the WRF model outputs in the inner domains is 5 km (5 × 10 × 5 × 10 = 2500). A surface area of 2500 km^2^ is chosen here because this is the closest surface area to 2400 km^2^ that may be attained when using a square as a searching region for the construction of IP Metrics 3 and 4 given the inner-domain model resolution of 5 km. As explained previously, 2400 km^2^ is the surface area of the drainage basin of the city of Asheville, NC, which was taken as the target area for the application of a physically based storm transposition method to four historical and two future TCs in Mure-ravaud [43].

**Figure 6:**
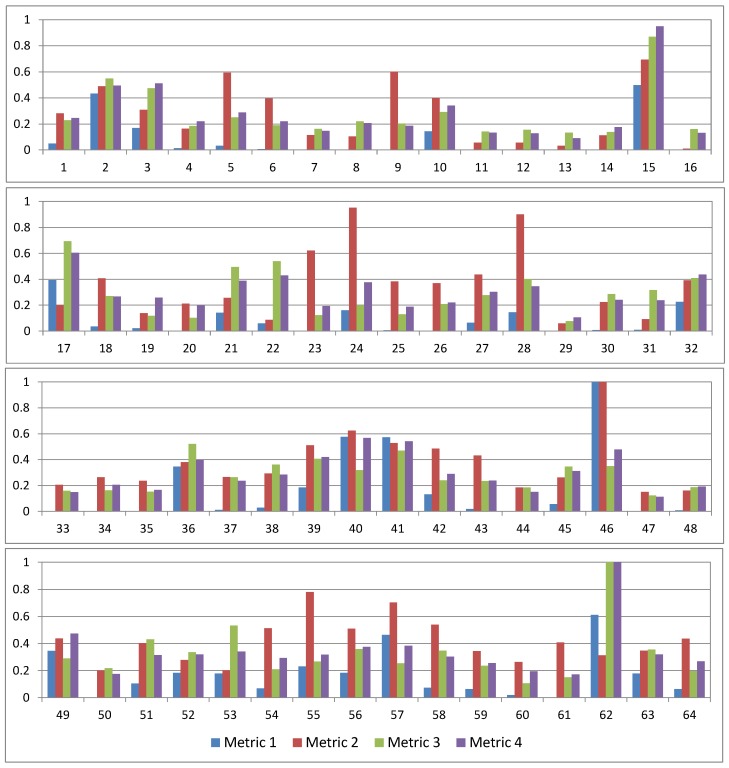
Normalized IP metrics for the 64 landfalling TCs found in the WRF model simulations. TCs are given chronologically. These normalized values were obtained by dividing the dimensional IP metrics by the maximum values given in Table 5. The dates of each TC may be found in Table A1.

Results are given in Figure 6. Note that, in this figure, IP metrics were normalized in order to be able to plot them next to each other. This normalization was performed by dividing each series by the maximum value of this series so that the maxima of the normalized series are equal to 1. The most extreme metric values that were used for the normalization are provided in Table 5.

**Figure A1:**
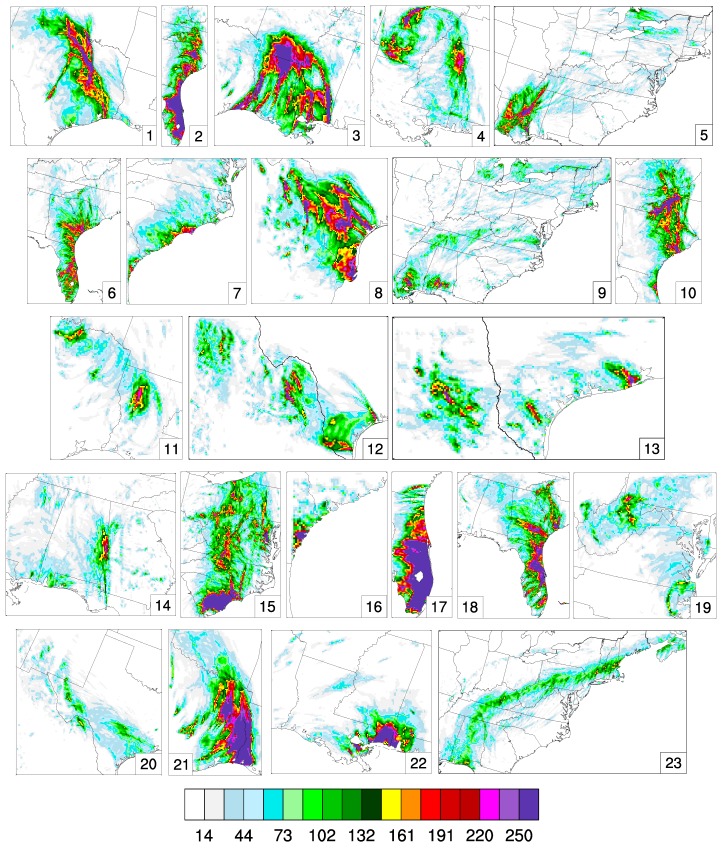

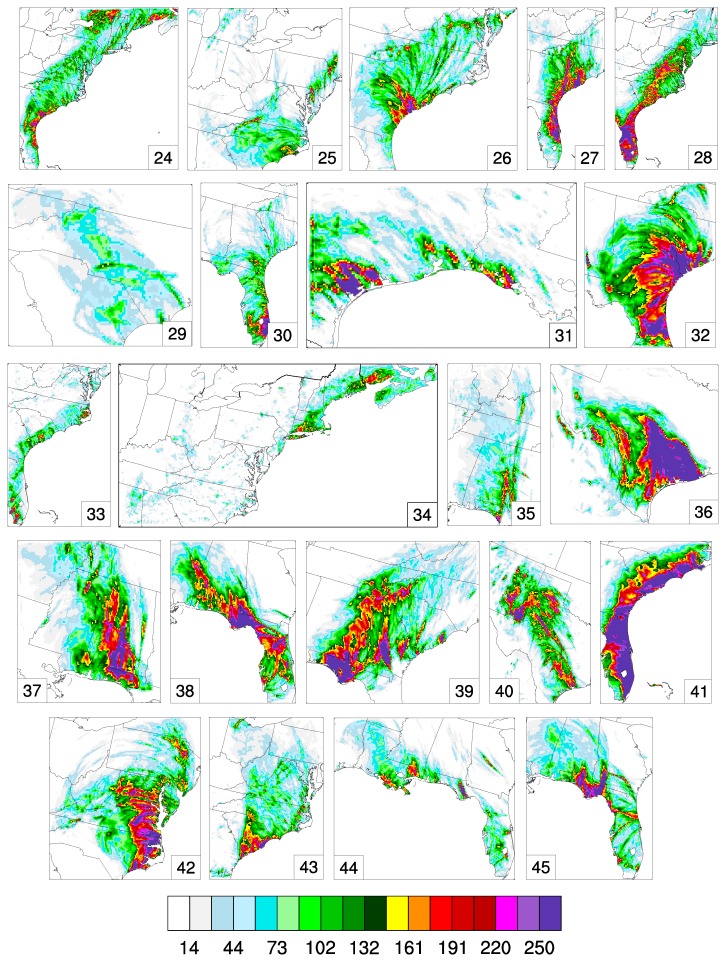

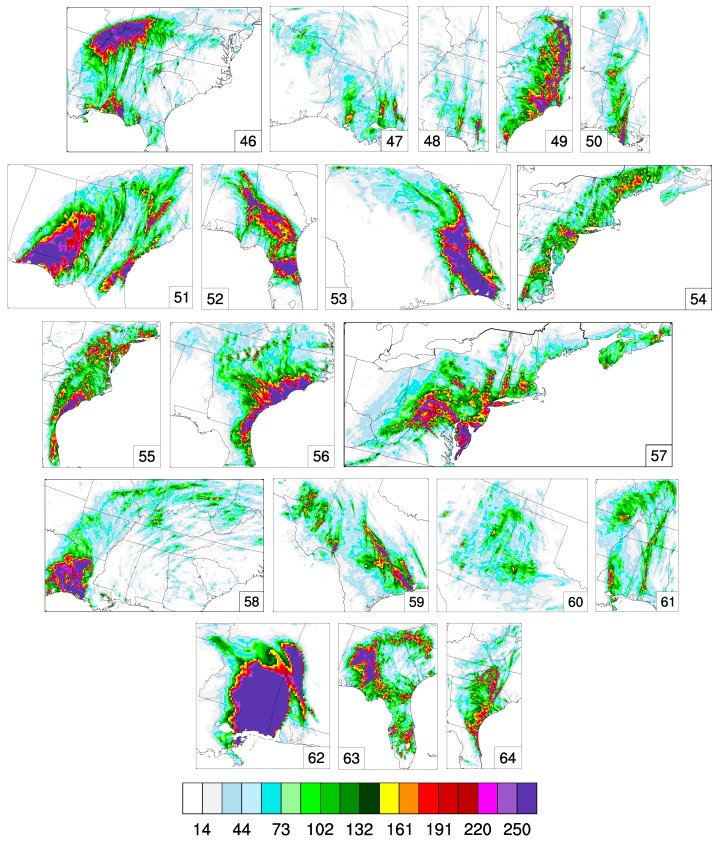
PD (mm) fields in simulation inner domains accumulated between the inner-domain simulation start and end dates provided in Table A1.

Figure 6 shows that, for certain TCs, the four normalized IP metrics take very different values. For example, for TC No. 23, IP Metric 1 is almost equal to 0, IP Metric 2 is relatively large (>0.6), and IP Metrics 3 and 4 are small but not negligible (>0.1 and ≤0.2). These results can be understood by examining the PD field of this TC shown in Figure A1 (Appendix B). This PD field is very large (which explains why the normalized IP Metric 2 is relatively large) as it is characterized by a long and narrow swathe affecting many states from eastern Mississippi, Alabama and northwestern Florida in the southern U.S. up to several states in New England. At the same time, the PD field is weak (which explains why the normalized IP Metric 1 is close to 0) except in a few locations (which explains why normalized IP Metrics 3 and 4 are not negligible) close to the location of landfall, in eastern Kentucky, and in New England.

**Figure 7:**
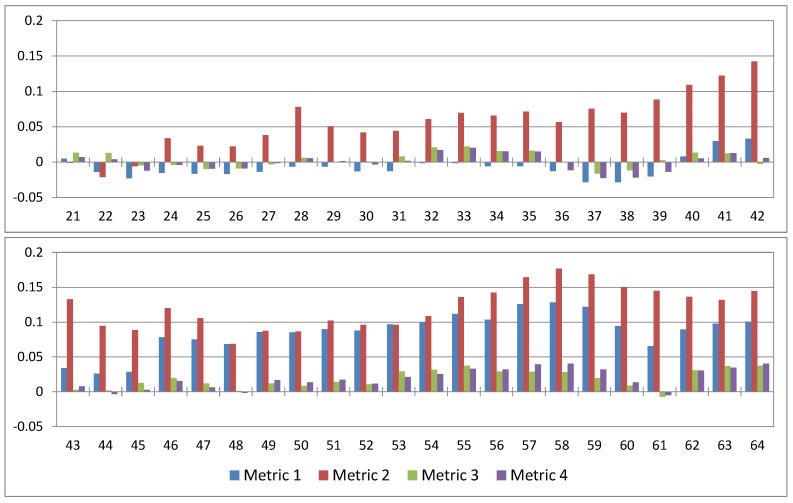
Anomalies of moving-average normalized IP metrics calculated using an averaging window of 20 TCs. These results were obtained from Figure 6 by performing the following two operations: (1) computing the twenty-TC moving average of the normalized metrics and (2) subtracting the average normalized metrics for TC No. 20 (which gives the mean of the normalized metrics of the first 20 landfalling TCs of the 21st century) from the others (which explains why the x-axis starts at TC No. 21). Positive values indicate an increase in IP compared to the first 20 TCs in the WRF simulations whereas negative values indicate a decrease in IP compared to the first 20 TCs in the WRF simulations.

Figure 7 presents the anomalies of the moving-average normalized IP metrics. It was constructed from Figure 6 by performing the following two operations: (1) computing the moving average of the results in Figure 6 using an averaging window of 20 TCs and (2) subtracting from the resulting averaged series the results for TC No. 20 representing the average normalized metrics for the first 20 landfalling TCs of the 21st century. Figure 7 helps to visualize how IP evolves throughout the 21st century. Indeed, positive values indicate an increase in IP compared to the first 20 TCs of the 21st century (note that TC No. 20 occurred in 2025, as shown in Table A1) whereas negative values indicate a decrease in IP.

It is seen that IP is expected to increase by the end of the century according to all metrics, although the increase is significantly larger for IP Metrics 1 and 2 than for IP Metrics 3 and 4. More precisely, this increase is the most pronounced for the anomaly of the moving-average normalized IP Metric 2 which becomes positive early, for TC No. 24 (in 2031), and remains positive until the end of the century. The moving-average normalized IP Metric 1 shows little variations (anomaly < 0.05 in absolute value) until TC No. 46 (in 2064) for which its anomaly exceeds 0.05 and remains significantly positive until the end of the century where it reaches a value of 0.1. The anomalies of the moving-average normalized IP Metrics 3 and 4 never exceed 0.05 in absolute value. They tend to be positive at the end of the century although they take slightly negative values for TC No. 61 (in 2089). These results suggest that, towards the end of the century, TCs may produce significantly larger and more intense PD fields. At the same time, their local IP (local meaning at the scale of a 2500 km^2^ region since it is the region size used for the construction of IP Metrics 3 and 4) may also increase, but more moderately.

## 7. Conclusions

This article presents a DD framework for the downscaling of a climate projection with the goal of investigating future IP from TCs. CCSM4 RCP 4.5 was used as the climate projection to provide IBCs to the WRF model. As is the case for many GCMs, CCSM4 does not contain a realistic population of TCs due to its coarse resolution, so that it was not appropriate to perform the direct DD of TCs from the GCM outputs. In this study, an alternative DD similar to the one used in Done et al. [16] was employed. It consists of using a large simulation outer domain in the hope that the RAM model will generate its own TCs. This approach was applied successfully to CCSM4 as the WRF model generated its own TCs in a large outer domain at 45 km horizontal resolution. These TCs were then detected and tracked in an automatized way using algorithms. Their properties including the CPD, MTSWS, RMTSWS, TCCHL, and TC depth were calculated and compared to TC properties in CFSR reanalysis dataset during the historical period 2005–2017. This comparison was the first step of a two-step validation process, and it aimed at ensuring that the model produced realistic TCs before pursuing DD to finer resolutions.

IP from TCs during the 21st century was then downscaled sequentially using one-way nesting. More precisely, the DD of the TCs in the WRF model outputs at 45 km resolution was first performed to 15 km resolution using a fixed intermediate domain encompassing all landfall locations. DD to 5 km resolution was not performed using a fixed inner domain because of the overwhelming computational effort associated with the simulation of the whole eastern and southern U.S. at 5 km resolution. Algorithms were created to construct the simulation inner domains for DD to 5 km resolution based on the PD fields in the intermediate domain. The aforementioned algorithms, including the algorithms for TC detection and tracking, and the algorithm for the construction of the simulation inner domains were developed to automatize the DD procedure as much as possible. Although such algorithms may not perform as well as if the tasks were performed manually, they offer the possibility to apply DD to many climate projections and/or to sets of model options with little manual action required, which would otherwise result in a tremendous workload.

Four metrics were proposed to quantify IP. These metrics were calculated for the simulated PD fields as well as the observed PD fields from Stage IV during the period 2005–2017. This was the second step of the aforementioned two-step validation process which aimed at ensuring that the model performed well in simulating IP from TCs during the historical period. The WRF model gave satisfactory performance, although the simulated PD fields tended to be spatially slightly too compact and locally too intense. The four IP metrics were then computed for the rest of the 21st century. It was found that IP from TCs may increase by the end of the century, although this increase concerns more the size and overall intensity of the PD fields (as measured by IP Metrics 1 and 2) than the local (basin scale) IP (as measured by IP Metrics 3 and 4). It is noted that the tendencies found in this study, either in terms of TC properties or TC IP in the future, should be treated with caution. Indeed, DD was performed only for one climate projection and one set of the WRF model options. Many more simulations are needed to legitimately draw any solid conclusion on such tendencies.

## Figures and Tables

**Table 1:** Parameterization schemes used for the dynamical downscaling (DD) of the Community Climate System Model version 4 (CCSM4) Representative Concentration Pathway (RCP) 4.5 projection with the Weather Research and Forecasting (WRF) model.

Parameterization	Name of the Scheme
**Microphysics**	WRF double moment 6-class (WDM6)
**Cumulus ^a^**	New Simplified Arakawa-Schubert (SAS)
**Planetary Boundary Layer**	Mellor-Yamada-Janjic (MYJ)
**Longwave Radiation**	Rapid Radiative Transfer Model (RRTM)
**Shortwave Radiation**	Dudhia
**Land Surface**	Unified Noah land-surface model
**Surface Layer**	Monin-Obukhov (Janjic Eta)

^a^ Cumulus parameterization was used in the outer and intermediate domains only.

**Table 2:** Number of TCs for three TC categories during the period 2004–2017 obtained using either observed data (column *Reported*) or the detection and tracking algorithms (column *Detected*). Observed data are from the free online encyclopedia *Wikipedia* (see for example https://en.wikipedia.org/wiki/2004_Atlantic_hurricane_season for the Atlantic hurricane season in 2004; last accessed on 18 February 2019). TS: tropical storm.

Year	TC Strength ≥ TS		TC Strength ≥ Hurricane		TC Strength = Major Hurricane (Category 3, 4 and 5)	
	Reported	Detected	Reported	Detected	Reported	Detected
2004	15	8	9	7	6	5
2005	28	12	15	10	7	6
2006	10	4	5	3	2	2
2007	15	1	6	1	2	1
2008	16	9	8	7	5	5
2009	9	4	3	3	2	2
2010	19	7	12	5	5	4
2011	19	6	7	5	4	3
2012	19	7	10	7	2	2
2013	14	3	2	1	0	0
2014	8	5	6	5	2	2
2015	11	5	4	4	2	2
2016	15	7	7	5	4	3
2017	17	6	10	6	6	5
average per year	15.4	6	7.4	4.9	3.5	3

**Table 3:** Mean value of TC properties in the WRF model outputs and in the Climate Forecast System Reanalysis (CFSR) for 2005–2017. CPD: central pressure deficit, MTSWS: maximum tangential surface wind speed, RMTSWS: radius of MTSWS.

TC Property	WRF Model Outputs	CFSR
	(45 km Resolution)	
CPD (mbar)	17.8	15.4
MTSWS (m s${}^{-1}$)	14.3	13.8
RMTSWS (km)	135	158
TCCHL (km)	387	467
TC depth (km)	11.0	12.9

**Table 4:** WRF model validation results for the simulation of IP during the period 2005–2017 measured by (a) the mean and (b) the standard deviation of IP Metrics 1 and 2. These parameters were computed by calculating the mean and standard deviation during the period 2005–2017 of the metrics of all landfalling TCs in the WRF model outputs (Column 2), of all historical landfalling TCs (the list of which is given in the supplementary material *Suppl.Mat._Historical_Landfalling_TCs.pdf*) with strength ≥ TS strength (Column 3), and of all historical landfalling TCs with strength ≥ hurricane strength (column 4).

**(a) Mean**	**WRF Model** **Outputs**	**Stage IV (Observation)**	
	**All TCs**	**TC Strength** **≥ TS Strength**	**TC Strength** **≥ Hurricane Strength**
IP Metric 1 (km^2^)	4.54 × 10^3^	3.12 × 10^3^	5.20 × 10^3^
IP Metric 2 (km^2^)	1.06 × 10^5^	1.43 × 10^5^	1.76 × 10^5^
**(b) StandardDeviation**	**WRF Model** **Outputs**	**Stage IV (Observation)**	
	**All TCs**	**TC Strength** **≥ TS Strength**	**TC Strength** **≥ Hurricane Strength**
IP Metric 1 (km^2^)	7.73 × 10^3^	7.01 × 10^3^	9.37 × 10^3^
IP Metric 2 (km^2^)	6.44 × 10^4^	8.74 × 10^4^	6.87 × 10^4^

**Table 5:** Most extreme values taken by the IP metrics based on the 64 landfalling TCs from the WRF model outputs.

Metric	Most Extreme	Obtained for TC No.
	Value	(See Table A1)
1	58.30 × 10^3^ km^2^	46
2	331.4 × 10^3^ km^2^	46
3	1152 mm	62
4	3.48	62

## Supplementary Materials

The following are available online at https://www.mdpi.com/1660-4601/16/5/687/s1.

## Abbreviations

The following abbreviations are used in this manuscript:

CCSM	Community Climate System Model
CCSM3	CCSM version 3
CCSM4	CCSM version 4
CFSR	Climate Forecast System Reanalysis
CLSLP	center of low sea level pressure
CPD	central pressure deficit
DD	dynamical downscaling
DTA	detection and tracking algorithm
GCM	general circulation model
GFDL	Geophysical Fluid Dynamics Laboratory
IBC	initial and boundary condition
IP	intense precipitation
MTSWS	maximum tangential surface wind speed
NCAR	National Center for Atmospheric Research
NCEP	National Centers for Environmental Prediction
NNRP	NCEP/NCAR Reanalysis Project
NOAA	National Oceanic and Atmospheric Administration
NRCM	Nested Regional Climate Model
PD	precipitation depth
PGW-DD	pseudo-global warming dynamical downscaling
PS	parameterization scheme
RAM	regional atmospheric model
RCP	Representative Concentration Pathway
RMTSWS	radius of maximum tangential surface wind speed
SLP	sea level pressure
SWS	surface wind speed
TC	tropical cyclone
TCCHL	TC characteristic horizontal length
TS	tropical storm
UTC	Coordinated Universal Time
WRF	Weather Research and Forecasting

## Appendix A. Simulation Start and End Dates for Dynamical Downscaling of the 64 Landfalling Tropical Cyclones to 15 km and 5 km Resolutions

Table A1 provides the simulation start and end dates for the intermediate and inner domains. The intermediate-domain simulation start date is the date for which the TC’s MTSWS, as computed in Section 4, was the largest as the storm moved over the Atlantic Ocean, whereas the end date was chosen so that the duration of the simulation of a TC in the intermediate domain is 2 weeks. For a given TC, the inner-domain simulation start date is one day before the time of landfall whereas the end date is the time of dissipation of the storm. Both of the inner-domain simulation start and end dates were estimated using algorithms which are presented in the supplementary material *Suppl.Mat._Creation_Inner_Domain.pdf*.

**Table A1:** Start dates and end dates for the simulation in the intermediate and inner domains of the 64 landfalling TCs. Coordinated Universal Time (UTC) is used.

Event #	Simulation Start Date for Intermediate Domain	Simulation End Date for Intermediate Domain	Simulation Start Date for Inner Domain	Simulation End Date for Inner Domain
1	08/11/2005 18:00	08/25/2005 18:00	08/14/2005 12:00	08/18/2005 12:00
2	08/27/2005 12:00	09/10/2005 12:00	08/29/2005 18:00	09/02/2005 18:00
3	08/14/2006 12:00	08/28/2006 12:00	08/17/2006 06:00	08/21/2006 06:00
4	08/12/2009 06:00	08/26/2009 06:00	08/14/2009 06:00	08/19/2009 12:00
5	09/10/2010 18:00	09/24/2010 18:00	09/13/2010 12:00	09/16/2010 12:00
6	08/02/2011 06:00	08/16/2011 06:00	08/04/2011 00:00	08/07/2011 00:00
7	08/22/2012 18:00	09/05/2012 18:00	08/24/2012 18:00	08/27/2012 18:00
8	08/26/2012 18:00	09/09/2012 18:00	08/30/2012 00:00	09/02/2012 00:00
9	08/13/2013 18:00	08/27/2013 18:00	08/17/2013 12:00	08/21/2013 12:00
10	08/28/2015 12:00	09/11/2015 12:00	08/30/2015 00:00	09/02/2015 18:00
11	08/28/2017 00:00	09/11/2017 00:00	08/31/2017 00:00	09/03/2017 18:00
12	07/30/2020 12:00	08/13/2020 12:00	08/05/2020 18:00	08/09/2020 12:00
13	09/15/2020 06:00	09/29/2020 06:00	09/20/2020 18:00	09/23/2020 18:00
14	08/07/2021 18:00	08/21/2021 18:00	08/10/2021 18:00	08/13/2021 18:00
15	08/15/2021 18:00	08/29/2021 18:00	08/20/2021 00:00	08/23/2021 18:00
16	08/27/2021 06:00	09/10/2021 06:00	08/28/2021 06:00	08/31/2021 06:00
17	10/11/2021 00:00	10/25/2021 00:00	10/15/2021 06:00	10/18/2021 06:00
18	09/03/2024 00:00	09/17/2024 00:00	09/04/2024 18:00	09/08/2024 00:00
19	09/10/2024 06:00	09/24/2024 06:00	09/15/2024 00:00	09/18/2024 00:00
20	08/22/2025 06:00	09/05/2025 06:00	08/25/2025 00:00	08/28/2025 06:00
21	09/03/2027 18:00	09/17/2027 18:00	09/07/2027 00:00	09/10/2027 18:00
22	09/02/2028 00:00	09/16/2028 00:00	09/07/2028 00:00	09/10/2028 00:00
23	08/19/2030 00:00	09/02/2030 00:00	08/21/2030 12:00	08/25/2030 18:00
24	08/29/2031 00:00	09/12/2031 00:00	09/02/2031 00:00	09/05/2031 12:00
25	09/23/2032 00:00	10/07/2032 00:00	09/27/2032 18:00	09/30/2032 18:00
26	08/26/2033 12:00	09/09/2033 12:00	08/28/2033 12:00	09/01/2033 00:00
27	09/03/2033 00:00	09/17/2033 00:00	09/04/2033 06:00	09/08/2033 00:00
28	09/21/2033 18:00	10/05/2033 18:00	09/23/2033 18:00	09/26/2033 18:00
29	09/04/2034 06:00	09/18/2034 06:00	09/07/2034 18:00	09/10/2034 18:00
30	07/17/2037 18:00	07/31/2037 18:00	07/19/2037 06:00	07/23/2037 00:00
31	09/23/2037 06:00	10/07/2037 06:00	09/26/2037 12:00	09/29/2037 12:00
32	08/30/2038 18:00	09/13/2038 18:00	09/02/2038 12:00	09/05/2038 12:00
33	09/03/2038 00:00	09/17/2038 00:00	09/08/2038 00:00	09/11/2038 00:00
34	07/12/2040 00:00	07/26/2040 00:00	07/19/2040 12:00	07/22/2040 12:00
35	08/21/2043 06:00	09/04/2043 06:00	08/24/2043 06:00	08/27/2043 06:00
36	08/26/2043 06:00	09/09/2043 06:00	09/02/2043 12:00	09/05/2043 12:00
37	08/20/2047 18:00	09/03/2047 18:00	08/25/2047 00:00	08/28/2047 00:00
38	08/24/2047 12:00	09/07/2047 12:00	09/04/2047 06:00	09/07/2047 12:00
39	09/23/2048 00:00	10/07/2048 00:00	09/29/2048 00:00	10/02/2048 00:00
40	08/31/2049 12:00	09/14/2049 12:00	09/03/2049 18:00	09/09/2049 06:00
41	09/26/2050 00:00	10/10/2050 00:00	09/27/2050 18:00	09/30/2050 18:00
42	08/28/2052 00:00	09/11/2052 00:00	08/31/2052 00:00	09/03/2052 12:00
43	08/30/2057 00:00	09/13/2057 00:00	09/01/2057 06:00	09/04/2057 12:00
44	09/27/2057 18:00	10/11/2057 18:00	10/01/2057 00:00	10/04/2057 00:00
45	09/09/2062 06:00	09/23/2062 06:00	09/12/2062 06:00	09/15/2062 06:00
46	09/13/2064 18:00	09/27/2064 18:00	09/17/2064 18:00	09/21/2064 12:00
47	08/16/2066 18:00	08/30/2066 18:00	08/19/2066 18:00	08/22/2066 18:00
48	08/22/2066 06:00	09/05/2066 06:00	08/24/2066 12:00	08/27/2066 12:00
49	08/26/2067 12:00	09/09/2067 12:00	08/30/2067 00:00	09/02/2067 06:00
50	08/12/2068 18:00	08/26/2068 18:00	08/16/2068 06:00	08/19/2068 18:00
51	08/24/2068 00:00	09/07/2068 00:00	08/29/2068 00:00	09/01/2068 00:00
52	08/29/2071 12:00	09/12/2071 12:00	09/01/2071 00:00	09/04/2071 00:00
53	09/17/2073 18:00	10/01/2073 18:00	09/23/2073 00:00	09/27/2073 00:00
54	08/04/2078 00:00	08/18/2078 00:00	08/06/2078 06:00	08/09/2078 06:00
55	09/07/2080 00:00	09/21/2080 00:00	09/11/2080 12:00	09/14/2080 18:00
56	09/18/2080 12:00	10/02/2080 12:00	09/22/2080 00:00	09/25/2080 00:00
57	09/24/2081 00:00	10/08/2081 00:00	10/02/2081 12:00	10/05/2081 12:00
58	09/14/2085 12:00	09/28/2085 12:00	09/20/2085 06:00	09/26/2085 00:00
59	08/18/2088 00:00	09/01/2088 00:00	08/22/2088 12:00	08/26/2088 12:00
60	08/30/2088 06:00	09/13/2088 06:00	09/02/2088 12:00	09/07/2088 06:00
61	09/13/2089 00:00	09/27/2089 00:00	09/16/2089 00:00	09/19/2089 00:00
62	09/17/2089 06:00	10/01/2089 06:00	09/22/2089 00:00	09/25/2089 06:00
63	08/16/2092 12:00	08/30/2092 12:00	08/18/2092 00:00	08/23/2092 12:00
64	08/18/2098 18:00	09/01/2098 18:00	08/25/2098 00:00	08/28/2098 00:00

## Appendix B. Plots of the Inner-Domain Precipitation Depth Fields of the 64 Landfalling Tropical Cyclones during the Period 2005–2100

This appendix presents the plots of the PD fields in the simulation inner domains accumulated between the inner-domain simulation start and end dates given in Table A1 for the 64 landfalling TCs simulated by the WRF model.
