# Supplementary material for: Investigation of Intense Precipitation from Tropical Cyclones during the 21st Century by Dynamical Downscaling of CCSM4 RCP 4.5
Source: Int J Environ Res Public Health. 2019 Feb 26;16(5):687. doi: 10.3390/ijerph16050687 (PMC6427206; doi:10.3390/ijerph16050687)
Supplement: Supplementary file 1 [file ijerph-16-00687-s001.zip › supplementary_files/Suppl.Mat._Application_DTAs_21st_century.pdf]

This supplementary document presents the results for the application of the detection and tracking algorithms (DTAs) to the WRF model outputs for the run at 45 km resolution in the simulation outer domain during 2005-2100. The number of tropical cyclones (TCs) per year from 2005 to 2100 is provided in Figure 1. A TC was considered as *landfalling* if its track reconstructed by the DTAs (it is reminded that these algorithms were only applied to grid points over the ocean) ended within 150 km from the conterminous U.S. coastline. It was checked manually for a few TCs that this criterion indeed allows distinguishing a landfalling TC from a non-landfalling TC.

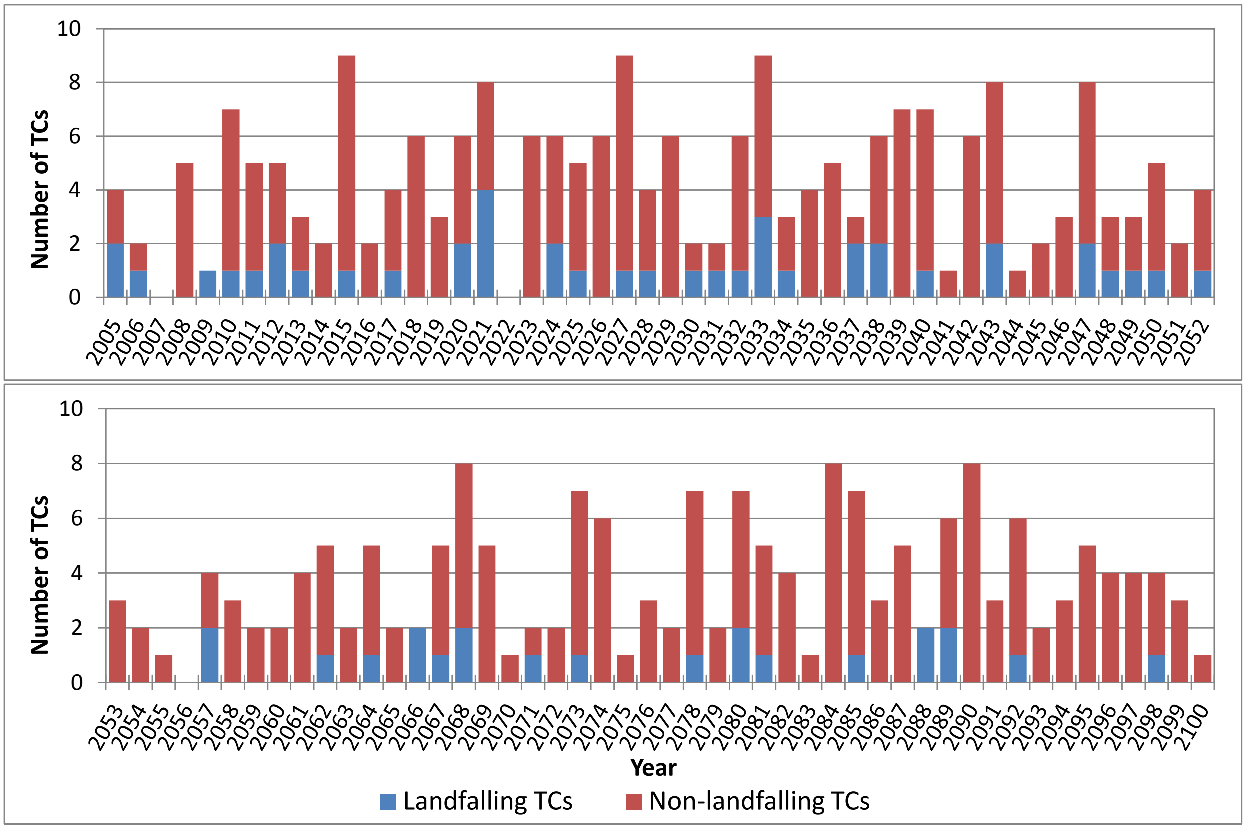

Figure 1: Number of landfalling and non-landfalling TCs detected in the WRF model outputs at 45 km resolution.

A total of 49 TCs were detected during 2005-2017, including 11 landfalling TCs. This is less than the number of TCs detected in CFSR (76 TCs were detected in CFSR during 2005-2017), but this still offers a sufficiently large population of TCs to work with. In particular, there was on average almost one landfalling TC per year during 2005-2017 in the WRF model outputs. Besides, 393 TCs were detected during 2005-2100 including 64 landfalling TCs.

Figure 2 shows the anomalies of the 20-yr moving-average number of TCs with respect to 2005-2024. This graph was constructed starting from Figure 1 by performing the following two operations: 1) the TC number series of Figure 1 was averaged using an averaging moving window of 20 years and 2) the average number of TCs during 2005-2024 was subtracted from the series resulting from Step 1. This is the reason why the series starts in 2025: since the value in 2024 after Step 1 gives the average number of TCs during the 20-yr period from 2005 (i.e. 2024 - 20 + 1) to 2024 , this will give 0 when subtracting the average number of TCs during 2005-2024 in Step 2.

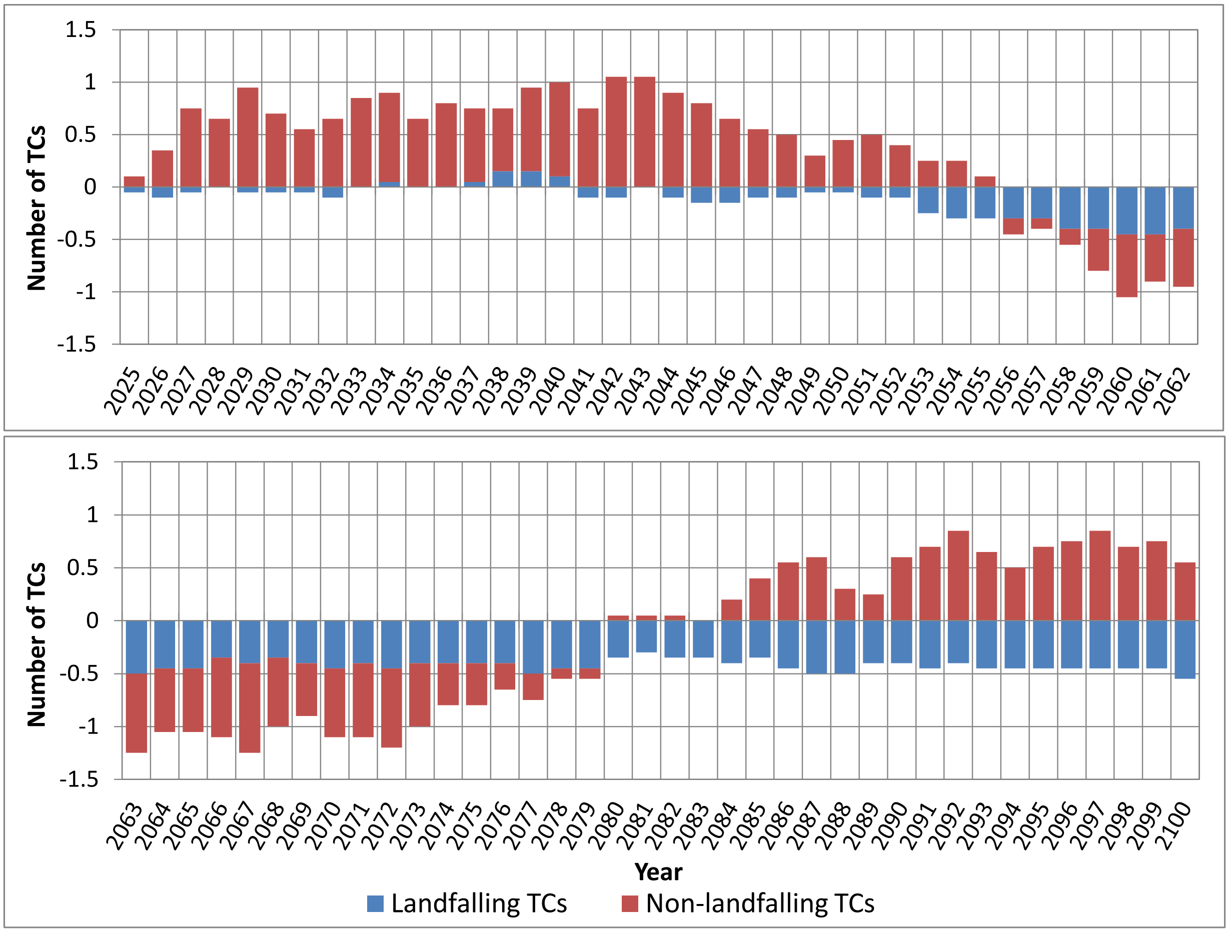

Figure 2: Anomalies with respect to 2005-2024 of the 20-yr moving-average number of landfalling and non-landfalling TCs detected in the WRF model outputs at 45 km resolution. These results were obtained from Figure 1 by performing the following two operations: 1) averaging the number of TCs using an averaging moving window of 20 years and 2) subtracting the average number of TCs for year 2024 from the other years. As a result, positive values indicate that the associated 20-yr period contains more TCs than 2005-2024 whereas negative values indicate that the associated 20-yr period contains less TCs than 2005-2024.

This graph helps visualizing how the number of landfalling and non-landfalling TCs changes throughout the 21st century with respect to the beginning of this century (i.e. 2005-2024). Three time periods can be identified in Figure 2. First, there is a 30-yr period (from 2025 to approximately 2055 in Figure 2) during which the WRF model produced overall more non-landfalling TCs but about the same number of landfalling TCs. Secondly, a 25-yr period is observed (from 2055 to 2080) during which the number of TCs (both landfalling and non-landfalling) identified in the WRF model outputs was significantly less than during 2005-2024, with about 1 TC less every year compared to 2005-2024. Finally, there is a 20-yr period at the end of the 21st century (from 2080 to 2100) when the number of non-landfalling TCs is again larger than during 2005-2024 whereas the number of landfalling TCs is smaller than during 2005-2024.
