# Supplementary material for: Investigation of Intense Precipitation from Tropical Cyclones during the 21st Century by Dynamical Downscaling of CCSM4 RCP 4.5
Source: Int J Environ Res Public Health. 2019 Feb 26;16(5):687. doi: 10.3390/ijerph16050687 (PMC6427206; doi:10.3390/ijerph16050687)
Supplement: Supplementary file 1 [file ijerph-16-00687-s001.zip › supplementary_files/Suppl.Mat._Creation_Inner_Domain.pdf]

This supplementary material describes the algorithms used to automatize the determination of the inner-domain simulation start and end dates, and the creation of the simulation inner domains for the dynamical downscaling (DD) to 5 km resolution of the precipitation depth (PD) fields of the tropical cyclones (TCs) simulated by the WRF model. These algorithms use the PD fields simulated in the intermediate domain at 15 km resolution. More precisely, in each case, the simulation inner domain is constructed based on the size and location of the TC's PD field in the intermediate domain.

It is reminded from the article that, in this study, a large intermediate domain encompassing the whole eastern and southern U.S. (Figure 1) was used because this allows to account for the diversity in TC tracks and the resulting diversity in landfall locations. At the same time, a resolution of 15 km remained coarse enough to make such simulations possible in a reasonable time. For a given TC, the start date for the simulation in the intermediate domain was taken as the date for which the maximum tangential surface wind speed was the largest as the TC was moving over the Atlantic Ocean. In addition, each simulation in the intermediate domain was performed for a duration of 2 weeks, as this is generally enough time for a TC to reach the U.S., make landfall, and dissipate.

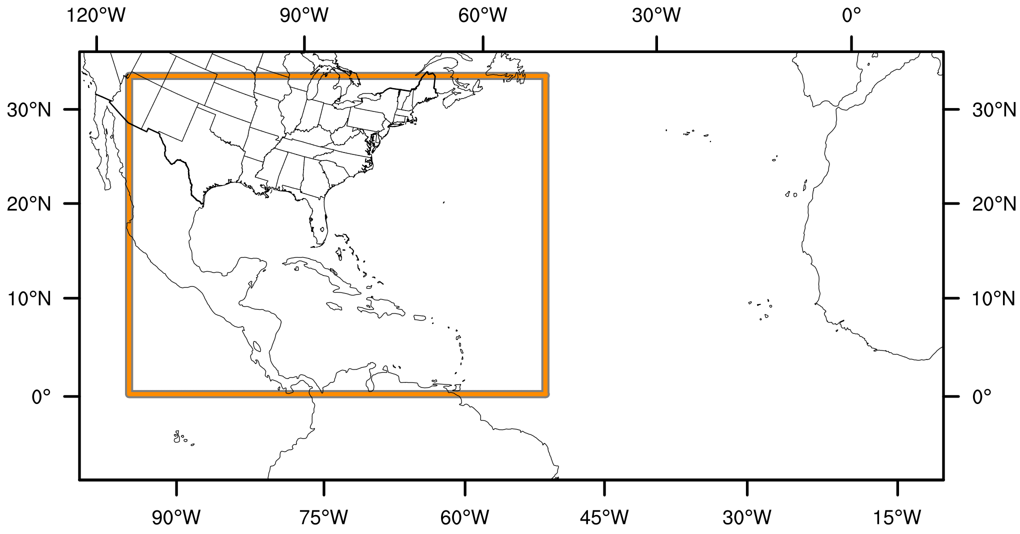

Figure 1: Simulation intermediate domain used for the DD to 15 km resolution. The intermediate domain is composed of 346 × 265 grid points.

As far as the simulation inner domain is concerned, using a large domain encompassing all landfall locations would be computationally challenging. Although such a computational exercise would have been feasible within the context of this article, because DD is performed for only one climate projection and one combination of the WRF model's parameterization schemes, it is reminded that one objective of this study is to propose an approach for the DD of precipitation from future TCs that may be applied massively to several climate projections and sets of model options within a reasonable time frame.

The proposed alternative consists in using the PD field in the intermediate domain to construct the inner domain and to determine the inner-domain simulation start and end dates, while being as conservative as possible: the inner domain should be as small as possible and the duration of the simulation as short as possible to reduce the time of computation. In fact, why didn't we construct

the simulation intermediate domain in the same way, using the PD field simulated in the outer domain at 45 km resolution? In the case of the DD in the intermediate domain, the simulation start date was taken relatively early so that a TC is usually still far from its location of landfall. As a result, because of the nonlinearity involved in the dynamics and thermodynamics of these storms, the change in resolution from 45 to 15 km can be enough to significantly modify the TC track, thus resulting in a different PD field over the U.S. On the other hand, if the inner-domain simulation start date is taken close enough from the time of landfall, there should not be any significant difference between the TC track simulated in the inner domain and the TC track simulated in the intermediate domain. In other words, if the inner-domain simulation start date is taken late enough, the inner domain established based on the location of the PD field in the intermediate domain shall contain the PD field simulated at 5 km resolution.

Determination of a TC track in the intermediate domain and of the time of landfall

The first step in the construction of the simulation inner domain for the DD of a given TC was to determine the track of this TC in the intermediate domain as well as the location and time of landfall. A TC track was already obtained in the outer domain, but as discussed previously, the track in the downscaled simulation at 15 km resolution may be different from the track in the simulation at 45 km resolution. The method used to determine the TC track in the intermediate domain was different from the method used in the outer domain, which is discussed in the supplementary material *Suppl.Mat._Detection&Tracking_algorithms.pdf*. In the outer domain, it was not known *a priori* where and when there is a TC in the WRF model outputs so that involved detection and tracking algorithms were used.

As far as the intermediate domain is concerned, it is known from the onset that there is a TC at the simulation start date, and the location of this TC is also known. The sea-level pressure (SLP) field was used to determine the TC track in the intermediate domain: starting from the location of the TC's center of low SLP (CLSLP) at the (intermediate-domain) simulation start date (i.e. at time step k = 0), the location of the CLSLP at time step k = 1 was obtained by moving towards lower values of the SLP field at time step k = 1 until a minimum is found, like a ball moving down along the sides of a bowl, similar to what was done to find the location of the CLSLP starting from the location of the barycenter in supplementary material *Suppl.Mat._Detection&Tracking_algorithms.pdf* (see Figure 4 in this document). This process was then repeated for the following time steps. Besides, the time step of 1 hour for the WRF model outputs was sufficiently small to allow the SLP field to change smoothly from one time step to another, thus offering a robust method to determine the TC track in the intermediate domain.

Special care was given to determine the time of landfall. Indeed, a TC can make landfall several times. In this case, which one to choose? A threshold for the duration spent on land was used to retain the most appropriate landfall. More precisely, the algorithm was stopped only if the TC spent more than 6 hours on land after landfalling, which allowed disregarding landfalling on the Caribbean islands and landfalling in Florida for those TCs which continue their journey in the Gulf of Mexico. The inner-domain simulation start date was then taken as the time of landfall minus one day.

Determination of the inner-domain simulation end date

The second step was to determine the inner-domain simulation end time. To do so, an algorithm was developed to determine the time of termination of the TC, defined as the time for which precipitation spawned by the TC in the U.S. becomes negligible. This task was more difficult than it seems because several regions of IP corresponding to different storms can coexist in the intermediate domain, but only one of them corresponds to the TC. The first attempt consisted in studying the time series of the intermediate domain-averaged precipitation. It failed for the aforementioned reason, emphasizing the need for a more sophisticated approach.

Eventually, the following approach was used. It consisted of 1) identifying all storms present in the intermediate domain at each time step, 2) determining which storm corresponds to the TC, and 3) determining the time of termination of the TC. Storms were identified in the following way. At each time step, the most intense part of the (hourly) PD field in the intermediate domain was determined by considering the grid points receiving a PD larger than a certain PD threshold. This PD threshold was taken as the 99.9^{th} percentile of the PD field. The next step was to regroup the grid points above the PD threshold into individual storms. To do so, a distance threshold was used: for each grid point P in the intermediate domain at which the PD threshold was exceeded, we looked around P to check if a storm had already been identified in the vicinity, within the distance given by the aforementioned threshold. If so, P was added to the ensemble of points forming the storm and the properties of this storm including the location of its barycenter, its minimum x-coordinate x_{min}, maximum x-coordinate x_{max}, minimum y-coordinate y_{min}, and maximum y-coordinate y_{max} (see Figure 2c) were recalculated taking the new point P into consideration. If not, a new storm was added to the list of storms at that time step; the new storm being composed of the point P only.

The distance between a grid point P and a preexisting storm may be defined in several ways (Figure 2). Certainly, the most legitimate approach is to calculate the distances between P and all the points composing the storm, and then to define the distance between P and the storm as the minimum of these distances (Figure 2a). However, this approach may be computationally too intensive, especially when the storm is large. A second approach which is much less computationally expensive is to define the distance between P and the storm as the distance between P and the storm's barycenter (Figure 2b). However, if the storm has a complicated shape, P may be far from the storm's barycenter, in which case it may be wrongly rejected from the ensemble of grid points composing the storm. A third approach is to define the distance between P and the storm as the minimum distance between P and the smallest rectangle containing the storm (Figure 2c). This approach is also computationally inexpensive and was retained in the present study. It generally provides satisfactory results, but it may lead in some cases to overestimate the size of the inner domain (see for example TCs No. 5, 9 and 34 in Figure A1 (Appendix B) of the article).

At this stage, a collection of storms is obtained at each time step in the intermediate domain. The TC is then determined using a distance threshold: starting at the time of landfall $k = 0$, we look around the location of landfall what is the closest storm at time step $k = 1$. If this storm is located within the distance threshold from the location of landfall it is said that this storm is the TC at time step $k = 1$, and the TC location at time step $k = 1$ is taken as the location of the storm's barycenter. This process is continued for the following time steps. The algorithm is terminated if one of these three conditions is met: 1) the PD corresponding to the 99.9^{th} percentile threshold in the intermediate domain is smaller than a given threshold taken as 1 mm, 2) it is not

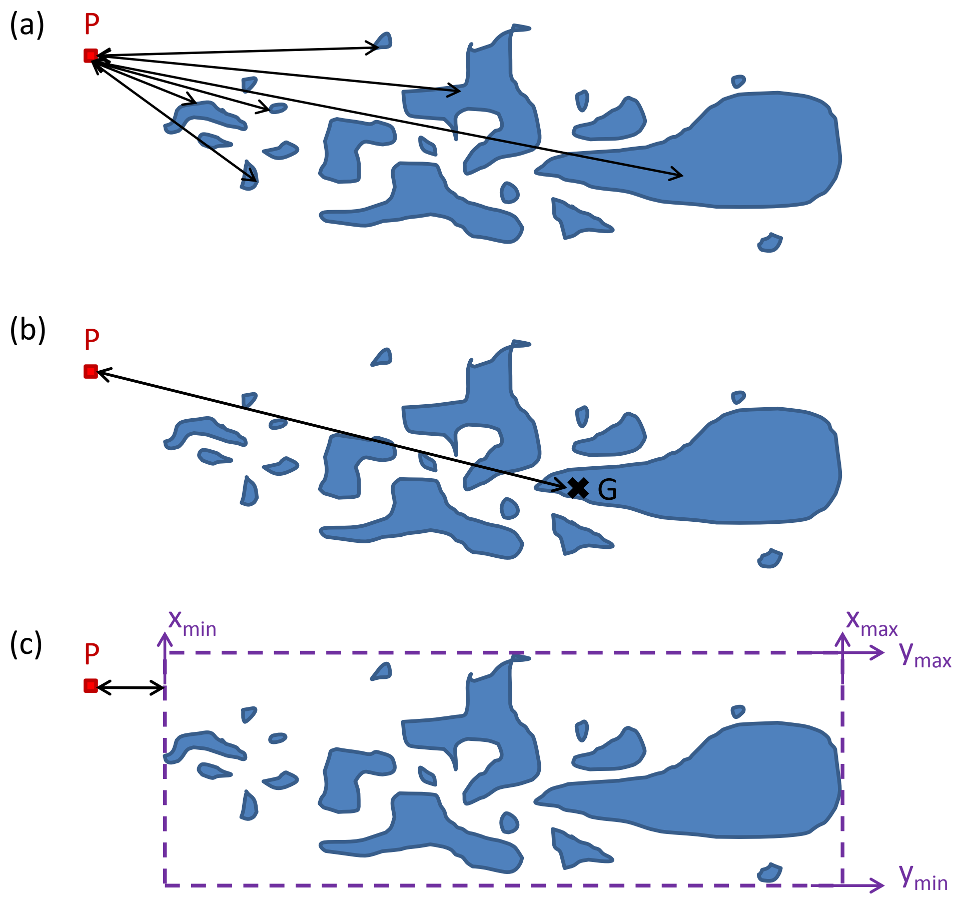

Figure 2: Three approaches to compute the distance between a point P (red square) and a storm (in blue; to be understood here as a relatively compact region of IP). (a) The distances between P and all the grid points constituting the storm are computed, and the distance between P and the storm is taken as the minimum of these distances. (b) the distance between P and the storm is taken as the distance between P and the storm's barycenter. (c) the distance between P and the storm is taken as the minimum distance between P and the smallest rectangle containing the storm.

possible to find a storm at the next time step which is sufficiently close to the TC, or 3) we reached the intermediate-domain simulation end date in which case the inner-domain simulation end date is taken as the intermediate-domain simulation end date. The first condition is used to make sure that only storms producing large enough PDs are considered.

At the end of the process described previously one obtains the inner-domain simulation end date. Eventually, if the inner-domain simulation end date was such that the duration of the simulation in the inner domain was less than 72 hours, it was modified so that the duration of the simulation equals 72 hours. This aims at making sure that the simulation in the inner domain is not too short so that one can at least compute a 72-h PD field.

Construction of the simulation inner domain

The third step was to determine the location and size of the inner domain. To do so, the PD field in the intermediate domain, accumulated between the inner-domain simulation start date and

the inner-domain simulation end date, was first computed. Afterwards, we used a similar approach as the one described previously. More precisely, a PD threshold was chosen to identify the most intense fraction of the PD field (red regions in Figure 3). In this case, good results were obtained using the 95th percentile. Using a larger threshold was generally too stringent, resulting in a too small inner domain. Grid points above the PD threshold were then gathered into storms based on a distance threshold by using the method described previously to compute the distance between a point and a storm. Eventually, the rectangle associated with the largest storm was retained as the simulation inner domain. Since the simulation start and end dates were selected to best capture the lifetime of the TC after landfall, the largest storm must correspond to the TC.

Results from this method are illustrated in Figure 4 for three TCs. It is observed that the method gives satisfactory results. Indeed, in each case, the simulation inner domain (represented by a black rectangle in Figure 4a, c and e) encompasses the PD field produced by the TC in the intermediate domain without being too large. Furthermore, the inland PD field in the left column, obtained by accumulating the PD field in the intermediate domain between the inner-domain simulation start and end dates, is about as intense as the inland PD field in the right column, obtained by accumulating the PD field in the intermediate domain between the intermediate-domain simulation start and end dates. This shows that the method performed well in determining the time interval during which precipitation from the TC fell in the U.S.

These efforts to reduce the size of the inner domains and the duration of the simulation in the inner domains by using algorithms to automatically detect where and when a given TC hit the country resulted in a drastic decrease of the computational effort associated with the WRF model simulations at the finest resolution of 5 km.

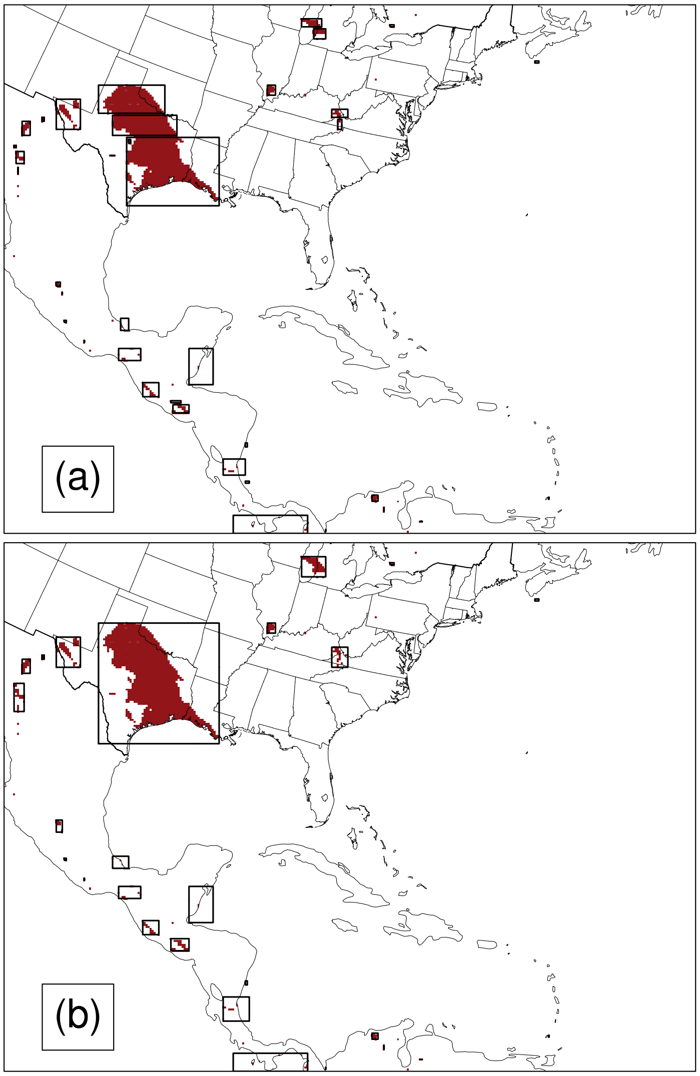

Figure 3: Illustration of the steps for the creation of the simulation inner domain for TC No. 1 in 2005 (see Table A1 in Appendix A of the article). Red regions correspond to the locations where the PD (accumulated from 08/14/2005 12:00 until 08/18/2005 12:00) exceeded the PD threshold taken as the 95th percentile of the PD field in the simulation intermediate domain. (a) shows the first step of the algorithm for creating the inner domain: grid points above the PD threshold (in red) are gathered based on a distance threshold to form regions of IP represented by black rectangles. Results from this step are sensitive to how grid points are sampled in the intermediate domain. In this example, the algorithm first iterated in the y-direction (i.e. the intermediate domain was sampled from South to North for a fixed x-location). (b) shows the final results: regions of IP from the first step in (a) are themselves combined to form storms based on a distance threshold. Eventually, the largest rectangle is taken as the inner domain, as can be verified in this example by comparing with TC No. 1 in Figure A1 of the article (Appendix B).

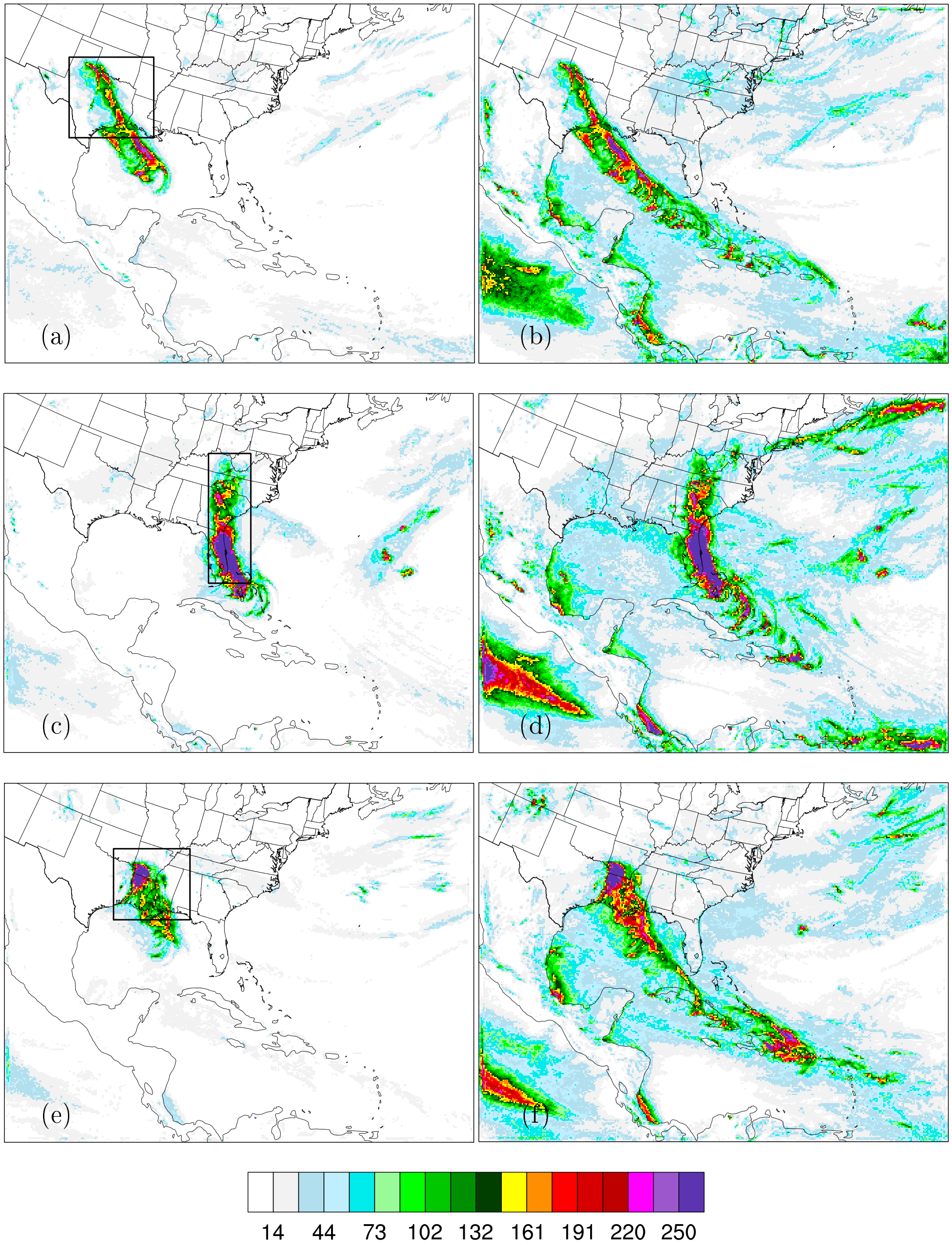

Figure 4: Validation of the algorithms for the set-up of the simulation inner domains by examining the first three TCs from the WRF model outputs (TCs No. 1, 2 and 3 in Appendices A and B of the article). (a)(b) correspond to TC No. 1, while (c)(d) are for TC No. 2, and (e)(f) are for TC No. 3. On the left, the **intermediate-domain** PD (mm) fields accumulated between the inner-domain simulation start and end dates (given by the rightmost two columns in Table A1) are provided along with the inner domains (black rectangles). On the right, the **intermediate-domain** PD fields accumulated between the intermediate-domain simulation start and end dates (given by the second and third columns in Table A1) are provided.
