# Supplementary material for: Investigation of Intense Precipitation from Tropical Cyclones during the 21st Century by Dynamical Downscaling of CCSM4 RCP 4.5
Source: Int J Environ Res Public Health. 2019 Feb 26;16(5):687. doi: 10.3390/ijerph16050687 (PMC6427206; doi:10.3390/ijerph16050687)
Supplement: Supplementary file 1 [file ijerph-16-00687-s001.zip › supplementary_files/Suppl.Mat._Detection_and_Tracking_algorithms.pdf]

This supplemental material describes the detection and tracking algorithms (DTAs) used to identify tropical cyclones (TCs) in the outputs of the WRF model for the run in the outer domain at 45 km horizontal resolution. Although TCs could have been identified manually, by visual examination of the model outputs, it was decided in this study to automatize these aspects so that the proposed dynamical downscaling (DD) procedure could be applied massively to many GCMs and/or many combinations of the model's options (e.g. parameterizations schemes) in a reasonable time.

1 Detection algorithm

The detection algorithm was adapted from Bengtsson et al. (1996). In that study, the ECHAM3[1] GCM was used at T106 resolution (i.e. a mesh with 320×160 nodes) in order to investigate the influence of greenhouse warming on the climatology of TCs. TCs in the model outputs were searched over the ocean, and were defined by the following physical and dynamical criteria:

1. Relative vorticity at 850 hPa $> 3.5 \times 10^{-5}$ s^{-1};
2. A maximum velocity of 15 m s^{-1} and a minimum surface pressure within a 7×7 grid point area around the point which fulfills condition 1;
3. The sum of the temperature anomalies (deviation from the mean, consisting of 7×7 grid points) for the levels 700, 500, and 300 hPa $> 3^{o}$C;
4. The temperature anomaly at 300 hPa > temperature anomaly at 850 hPa;
5. The mean wind speed at 850 hPa > mean wind speed at 300 hPa.

These criteria were found to be too stringent for the detection of TCs, both in the WRF model outputs and in the Climate Forecast System Reanalysis (CFSR; used for the validation of these algorithms). They were consequently modified by choosing lower threshold values. More specifically, two sets of criteria were used. The first set called *high-threshold* (HT) set allowed the detection of intense TCs, whereas the second set called *low-threshold* (LT) set was used for the reconstruction of their tracks. The HT set of criteria was:

1. Relative vorticity at 850 hPa $> 3 \times 10^{-5}$ s^{-1};
2. A maximum velocity of 14 m s^{-1} and a minimum surface pressure within a 7×7 grid point area around the point which fulfills condition 1;
3. The sum of the temperature anomalies (deviation from the mean, consisting of 7×7 grid points) for the levels 700, 500, and 300 hPa $> 1.5^{o}$C;
4. The temperature anomaly at 300 hPa > temperature anomaly at 850 hPa;
5. The mean wind speed at 850 hPa > mean wind speed at 300 hPa.

On the other hand, the LT set of criteria was:

[1]ECHAM is an atmospheric GCM, developed at the Max Planck Institute for Meteorology (`http://www.mpimet.mpg.de/en/science/models/echam.html`; last accessed 02/19/2019)

1. Relative vorticity at 850 hPa > 2×10^{-5} s^{-1};
2. A maximum velocity of 12 m s^{-1} and a minimum surface pressure within a 7 × 7 grid point area around the point which fulfills condition 1;
3. The sum of the temperature anomalies (deviation from the mean, consisting of 7 × 7 grid points) for the levels 700, 500, and 300 hPa > 0.5°C;
4. The temperature anomaly at 300 hPa > temperature anomaly at 850 hPa;
5. The mean wind speed at 850 hPa > mean wind speed at 300 hPa.

The search domain (i.e. the region where the grid points meeting the previous criteria were searched for) was taken as the band between 120°W and 15°W in longitude, and between 0°N and 45°N in latitude. Besides, the Caribbean Islands were added to the search domain because it was observed that if we limit the search to the ocean grid points as in Bengtsson et al. (1996), some TC tracks end on these islands whereas they actually just pass over the islands and keep going over the ocean afterwards, which is prejudicial when looking for TCs making landfall in the conterminous U.S.

After applying these criteria to the data, two collections of points were obtained at every time step: one for the HT set of criteria, and a larger one for the LT set of criteria. TCs were then identified from these collections of points based on a distance threshold of 550 km (Figure 1).

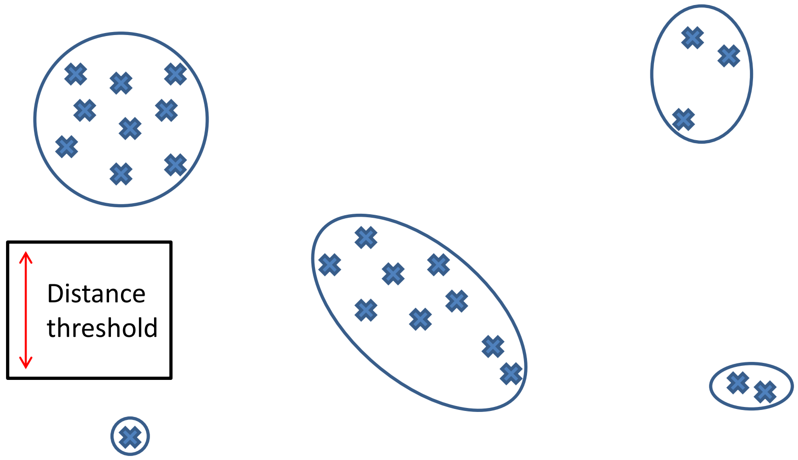

Figure 1: Illustration of the method to identify TCs (represented by ellipses in this figure) starting from the collection of grid points (represented by crosses) that pass the criteria at a given time step: adjacent grid points are grouped together based on a distance threshold of 550 km.

For the moment, the center of each TC is taken as the barycenter of the collection of points that constitute this cyclone. Indeed, finding the center of low sea-level pressure (CLSLP) requires additional effort, which will be done later. Thus, at this point, two collections of TCs are obtained from the two collections of grid points: one corresponding to the HT set of criteria and one corresponding to the LT set of criteria. Of course, the TCs associated with the HT set are included into the collection of TCs associated with the LT set (since the LT set uses less stringent criteria).

Finally, in order to retain only the most intense TCs and eliminate feeble vortices from the two populations, a threshold on the number of grid points constituting each vortex was applied. A threshold of 6 points was used in this study. For example, in Figure 1, only two of the TCs would be retained: the one with 9 grid points at the top left, and the one with 9 grid points in the middle.

2 Tracking algorithm

After using the detection algorithm, two populations of TCs are obtained at each time step: one for the HT set of criteria and one for the LT set of criteria. The following step was to match TCs at a given time step with TCs at the adjacent time steps. In other words, considering two TCs from two adjacent time steps, how does one know if they are the same storm or different storms?

To answer this question, a threshold noted d_{max} on the maximum distance that a TC can cover in a given time step was used. In other words, a threshold was put on the maximum TC speed (more precisely on the speed of the TC's barycenter). Considering a time step k and a TC i from the HT collection of TCs at that time step, we then looked in the LT collection of TCs at time step $k+1$ (k-1) for the TC j that was the closest from i spatially. If j happened to be located at a distance less than d_{max} away from i, then it was said that i and j are actually the same TC, and we moved on to the next (previous) time step $k+2$ (k-2) to see if a TC from the LT collection at time step $k+2$ (k-2) was located at a distance less than d_{max} away from j. The process was terminated when no TC from the LT collection could be found in the next (previous) time step that was located less d_{max} away from j. This process is illustrated in Figure 2 for the simpler case of a TC moving in a one-dimensional space (i.e. along a line). It is noted that the LT collection of TCs was used instead of the HT collection to reconstruct a TC's track because using only the HT collection would result in an incomplete track, since the HT criteria are not met during the early and late stages of a TC's lifetime.

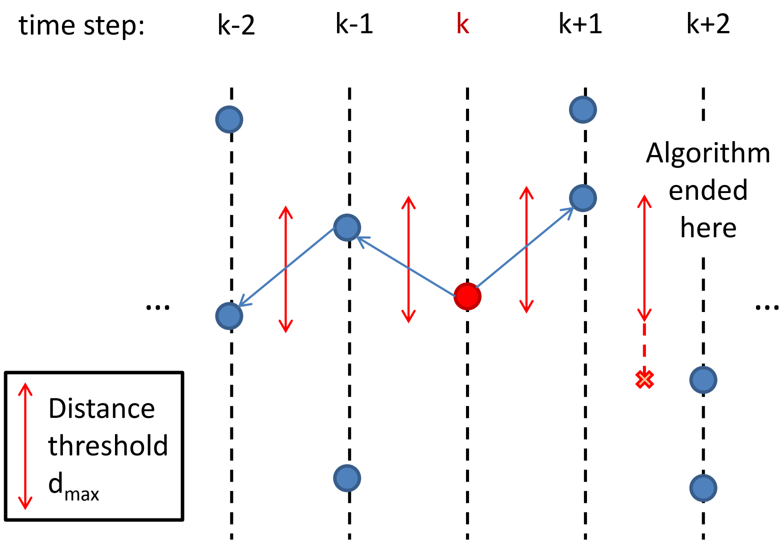

Figure 2: Illustration of the tracking algorithm for a one-dimensional space (i.e. TCs moving on a line). Starting from a given TC in the HT collection (red point) at time step k, we go forward and backward in time checking if there is a TC in the LT collection (blue points) that is located within a distance less than the distance threshold. If not, the algorithm is terminated (which happens at time step k+2 in this example).

At this stage, a track is obtained for each TC in the HT collection and for each time step. As a result, if a TC exists for N time steps in the HT collection, there will N duplicates of the same track and only one of them can be retained. In the end, a collection of TC tracks is obtained. Satisfactory results were obtained using a threshold of 600 km during 6 h for CFSR (which is provided with a time resolution of 6 h), and a threshold of 400 km during 1 h for the WRF model outputs (being produced with a time resolution of 1 h). The choice of the threshold values may seem surprising because the corresponding threshold on the TC speed is significantly larger in the case of the WRF model outputs (400 km h^{-1} vs. 100 km h^{-1} for CFSR). This is because, at this stage in the process, the barycenter of the collection of grid points that constitute a TC (Figure 1) is taken as the center of this TC, and there can be significant changes in the location of this barycenter from one time step to the next. This issue is illustrated in Figure 3. In other words, a TC track constructed using the locations of the barycenter is less smooth than a track constructed using the locations of the CLSLP, and this behavior is more evident in the hourly case (WRF model outputs) than in the 6-hourly case (CFSR). In fact, it was observed that, if a threshold of 100 km h^{-1} is used to process the WRF model outputs, TC tracks were generally incomplete because the algorithm finished too early.

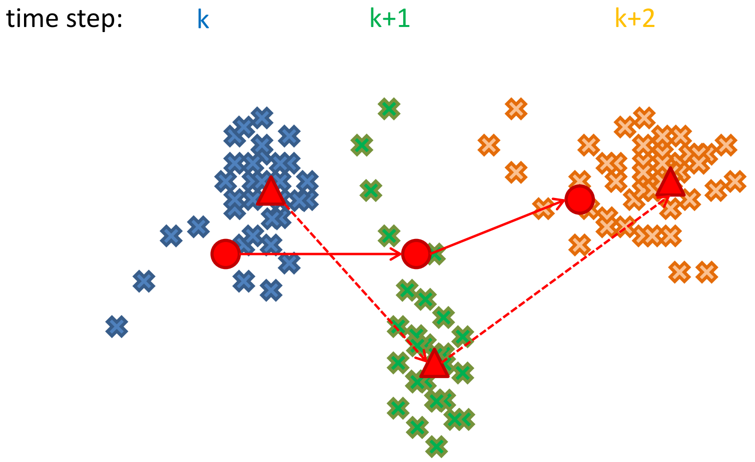

Figure 3: Illustration of the fact that TC tracks constructed using the barycenter (red triangle) are less smooth than TC tracks constructed using the CLSLP (red dots). Different colors show the same storm at different time step as it moves from left to right.

Besides, only TC tracks lasting for more than 72 h and starting at least 1500 km away from the conterminous U.S. coastline were retained in order to eliminate ephemeral systems from the TC population. It is noted that all these restrictions including the different thresholds used in the detection process may lead to miss a few TCs. This would be problematic if the goal of this study was to investigate the TC climatology. However, the article mainly focuses on the simulation of inland intense precipitation from TCs during the 21st century, so that, as long as the DTAs produce a sufficiently large population of TCs to work with, it is less problematic if they do not detect all TCs in the model outputs. In fact, the different restrictions and threshold values were decided so that the DTAs are loose enough to detect as many TCs as possible but conservative enough to limit unintended postprocessing work to remove manually spurious storms from the TC population.

The last step of the tracking process was to determine the location of the TC CLSLP to obtain smoother TC tracks. The process used to find the CLSLP is illustrated in Figure 4: starting from

the barycenter of the cloud of high-vorticity points (Figure 1), we moved down along the sea-level pressure (SLP) field until a local minimum was found. This SLP local minimum is the TC CLSLP. This method to locate the CLSLP generally works very well. However, in some cases, especially for weak TCs, the iterations lead to another local minimum of the SLP field which is not the TC CLSLP. Thresholds on the maximum number of descent iterations and maximum distance from the barycenter were used to avoid this issue: in the case one of these two thresholds is exceeded, the barycenter is kept as an estimate of the TC CLSLP.

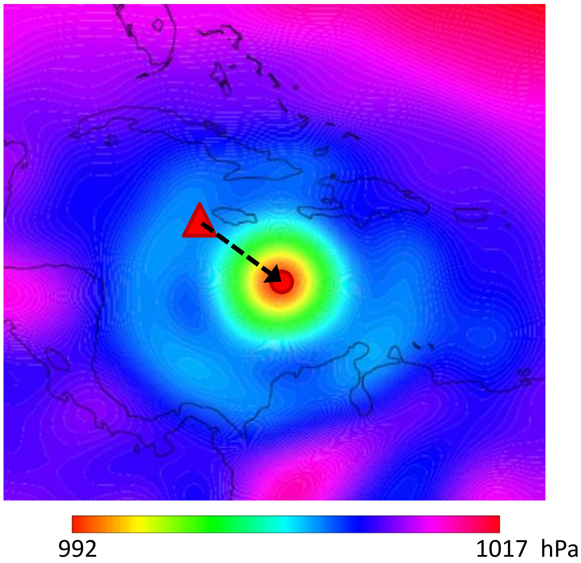

Figure 4: Illustration of the method used to find the CLSLP: starting from the barycenter (red triangle) identified in the previous steps, we move down along the SLP field (color plot) until the CLSLP (red circle) is found.

References

Bengtsson, L., Botzet, M., and Esch, M. (1996). “Will greenhouse gas-induced warming over the next 50 years lead to higher frequency and greater intensity of hurricanes?” In: *Tellus A* 48.1, pp. 57–73. DOI: 10.1034/j.1600-0870.1996.00004.x.
