# Supplementary material for: Investigation of Intense Precipitation from Tropical Cyclones during the 21st Century by Dynamical Downscaling of CCSM4 RCP 4.5
Source: Int J Environ Res Public Health. 2019 Feb 26;16(5):687. doi: 10.3390/ijerph16050687 (PMC6427206; doi:10.3390/ijerph16050687)
Supplement: Supplementary file 1 [file ijerph-16-00687-s001.zip › supplementary_files/Suppl.Mat._Historical_Landfalling_TCs.pdf]

This supplementary document provides the list of the historical landfalling tropical cyclones (TCs) during 2005-2017. TCs were determined by visual examination of TC tracks provided by the free online encyclopedia *Wikipedia* (by checking if these tracks passed over the conterminous U.S.), whereas the dates and times of landfall and termination were obtained from the Tropical Cyclones Reports of the National Hurricane Center. In Section 6 of the article, four intense precipitation (IP) metrics are computed for each of these TCs using the Stage IV observation dataset (Lin and Mitchell, 2005), and their statistics are compared with those corresponding to the TCs simulated by the WRF model during 2005-2017, the objective being to check if the WRF model produced realistic IP during the historical period.

Table 1: List of the historical landfalling TCs with strength $\geq$ TS during 2005-2017 with the dates and times of their formation, termination and first landfall in the conterminous U.S. This list was created by examining TC tracks obtained from the free online encyclopedia *Wikipedia* and by checking if these tracks passed over the conterminous U.S. Dates and times of formation, termination and landfall were then obtained from the Tropical Cyclones Reports of the National Hurricane Center (`https://www.nhc.noaa.gov/data/tcr/`; last accessed on 02/19/2019).

Name	Year	Formed	Ended	Landfall
TS Arlene	2005	06/08 18:00	06/14 12:00	06/11 19:00
Hurricane Cindy	2005	07/03 18:00	07/11 12:00	07/06 03:00
Hurricane Dennis	2005	07/04 18:00	07/18 12:00	07/10 19:30
Hurricane Katrina	2005	08/23 18:00	08/31 12:00	08/25 22:30
Hurricane Rita	2005	09/18 00:00	09/26 06:00	09/24 07:40
TS Tammy	2005	10/05 06:00	10/07 06:00	10/05 23:00
Hurricane Wilma	2005	10/15 18:00	10/27 00:00	10/24 10:30
TS Alberto	2006	06/10 06:00	06/19 12:00	06/13 16:30
Hurricane Ernesto	2006	08/24 18:00	09/04 12:00	08/30 03:00
TS Barry	2007	05/31 00:00	06/05 18:00	06/02 14:00
TS Erin	2007	08/15 00:00	08/20 00:00	08/16 10:30
TS Gabrielle	2007	09/08 00:00	09/11 12:00	09/09 15:30
Hurricane Humberto	2007	09/12 06:00	09/14 18:00	09/13 07:00
Hurricane Dolly	2008	07/20 12:00	07/27 06:00	07/23 18:20
TS Edouard	2008	08/03 12:00	08/07 00:00	08/05 12:00
TS Fay	2008	08/15 12:00	08/28 12:00	08/18 20:30
Hurricane Gustav	2008	08/25 00:00	09/05 18:00	09/01 15:00
Hurricane Hanna	2008	08/28 00:00	09/08 18:00	09/06 07:20
Hurricane Ike	2008	09/01 06:00	09/15 18:00	09/13 07:00
TS Claudette	2009	08/16 06:00	08/18 00:00	08/17 05:30
Hurricane Ida	2009	11/04 06:00	11/11 12:00	11/10 12:00
TS Bonnie	2010	07/22 06:00	07/26 00:00	07/23 14:30
TS Hermine*	2010	09/04 18:00	09/10 06:00	09/07 02:00
TS Don	2011	07/27 06:00	07/30 12:00	07/30 02:30
Hurricane Irene	2011	08/21 00:00	08/30 06:00	08/27 12:00
TS Lee	2011	09/02 00:00	09/07 00:00	09/04 10:30
TS Beryl	2012	05/25 12:00	06/02 06:00	05/28 04:10
TS Debby	2012	06/23 12:00	06/27 18:00	06/26 21:00
Hurricane Isaac	2012	08/20 12:00	09/01 12:00	08/29 00:00
Hurricane Sandy	2012	10/21 18:00	10/31 18:00	10/29 23:30
TS Andrea	2013	06/05 18:00	06/09 00:00	06/06 22:00

Hurricane Arthur	2014	06/28 18:00	07/10 00:00	07/04 03:15
TS Ana	2015	05/06 06:00	05/13 00:00	05/10 10:00
TS Bill	2015	06/16 00:00	06/21 06:00	06/16 16:45
TS Bonnie	2016	05/27 06:00	06/10 00:00	05/29 12:30
TS Colin	2016	06/05 12:00	06/09 00:00	06/07 02:00
Hurricane Hermine	2016	08/28 18:00	09/09 00:00	09/02 05:30
TS Julia	2016	09/13 06:00	09/21 12:00	09/13 06:00
Hurricane Matthew	2016	09/28 12:00	10/10 00:00	10/08 15:00
TS Cindy	2017	06/19 18:00	06/24 12:00	06/22 07:00
TS Emily	2017	07/30 18:00	08/02 06:00	07/31 14:45
Hurricane Harvey	2017	08/16 06:00	09/02 18:00	08/26 03:00
Hurricane Irma	2017	08/30 00:00	09/13 18:00	09/10 13:00
Hurricane Nate	2017	10/03 12:00	10/11 06:00	10/08 00:00

* TS Hermine actually made landfall on the coast of northeastern Mexico, close to the Texas boarder.

References

Lin, Y. and Mitchell, K. E. (2005). "The NCEP stage II/IV hourly precipitation analyses: Development and applications". In: *19th Conf. Hydrology, American Meteorological Society, San Diego, CA, USA*.
