# Supplementary material for: Investigation of Intense Precipitation from Tropical Cyclones during the 21st Century by Dynamical Downscaling of CCSM4 RCP 4.5
Source: Int J Environ Res Public Health. 2019 Feb 26;16(5):687. doi: 10.3390/ijerph16050687 (PMC6427206; doi:10.3390/ijerph16050687)
Supplement: Supplementary file 1 [file ijerph-16-00687-s001.zip › supplementary_files/Suppl.Mat._TC_properties_21st_century.pdf]

In Section 4 of the article, the methods used to calculate the properties of tropical cyclones (TCs) are first described. These properties include the central pressure deficit (CPD), the maximum tangential surface wind speed (MTSWS), the radius of MTSWS (RMTSWS), the TC characteristic horizontal length (TCCHL), and the TC depth. Secondly, these methods are applied to all the TCs identified in the Climate Forecast System Reanalysis (CFSR) and in the WRF model outputs for the run in the simulation outer domain at 45 km resolution during 2005-2017. The mean TC properties are then calculated for both cases and compared in order to assess whether the WRF model produced realistic TCs during the historical period in terms of the aforementioned TC properties. It was found that the WRF model produced a realistic population of TCs. Indeed, although TCs in the WRF model outputs tended to be slightly more intense and smaller in size compared to TCs in CFSR, this difference may be explained by the horizontal resolution which is slightly coarser for CFSR.

In this supplementary material, TC properties are calculated for the rest of the 21$^{st}$ century to investigate their evolution in the future. Figure 1 shows the evolution during the 21$^{st}$ century of the moving-average TC properties obtained using an averaging window of 20 years, and normalized so that TC properties range between 0 and 1. TC properties were normalized using the values shown in Table 1, and the dimensional series may be recovered by multiplying the normalized series by the associated maxima given in this table.

Table 1: TC property values used to normalize the series presented in Figure 1 (so that the maxima are equal to 1 for all TC properties). These are 20-yr averages, and do not correspond to instantaneous maxima attained by individual TCs.

| TC property | Maximum value |
|---|---|
| CPD | 18.10 mbar |
| MTSWS | 14.09 m s$^{-1}$ |
| RMTSWS | 144.9 km |
| TCCHL | 384.2 km |
| TC depth | 11.78 km |

Upon examining the ranges of values exhibited by the y-axes in Figure 1, it is observed that the variations of (20-yr moving-average) TC properties during the 21$^{st}$ century are relatively small: about 20% for the CPD, 10% for the MTSWS and RMTSWS, 5% for the TCCHL, and 25% for the TC depth. Nonetheless, some tendencies can be identified. First, it is observed that the variations of the CPD closely match the variations of the MTSWS. Three periods are observed in these two series: 1) a 25-yr period (from 2025 to approximately 2050 in Figure 1a and b) for which TCs tend to be slightly weaker than during 2005-2024, 2) a 20-yr period (from 2050 to 2070) for which TCs tend to be more intense than during 2005-2024, and 3) a 30-yr period (from 2070 to 2100) for which TCs tend to be slightly weaker than during 2005-2024. This reminds us of the three time periods identified in the supplementary material *Suppl.Mat._Application_DTAs_21st_century.pdf* when examining the evolution of the number of landfalling and non-landfalling TCs during the 21$^{st}$ century, although the time intervals do not match exactly.

Thus, the results seem to suggest that there may be in the future a period with overall more and weaker TCs, followed by a period with less but more intense TCs in the middle of the 21$^{st}$ century, and finally a period with slightly more and weaker TCs at the end of the century. These results differ from the findings of most numerical studies reported in the literature to the extent that such

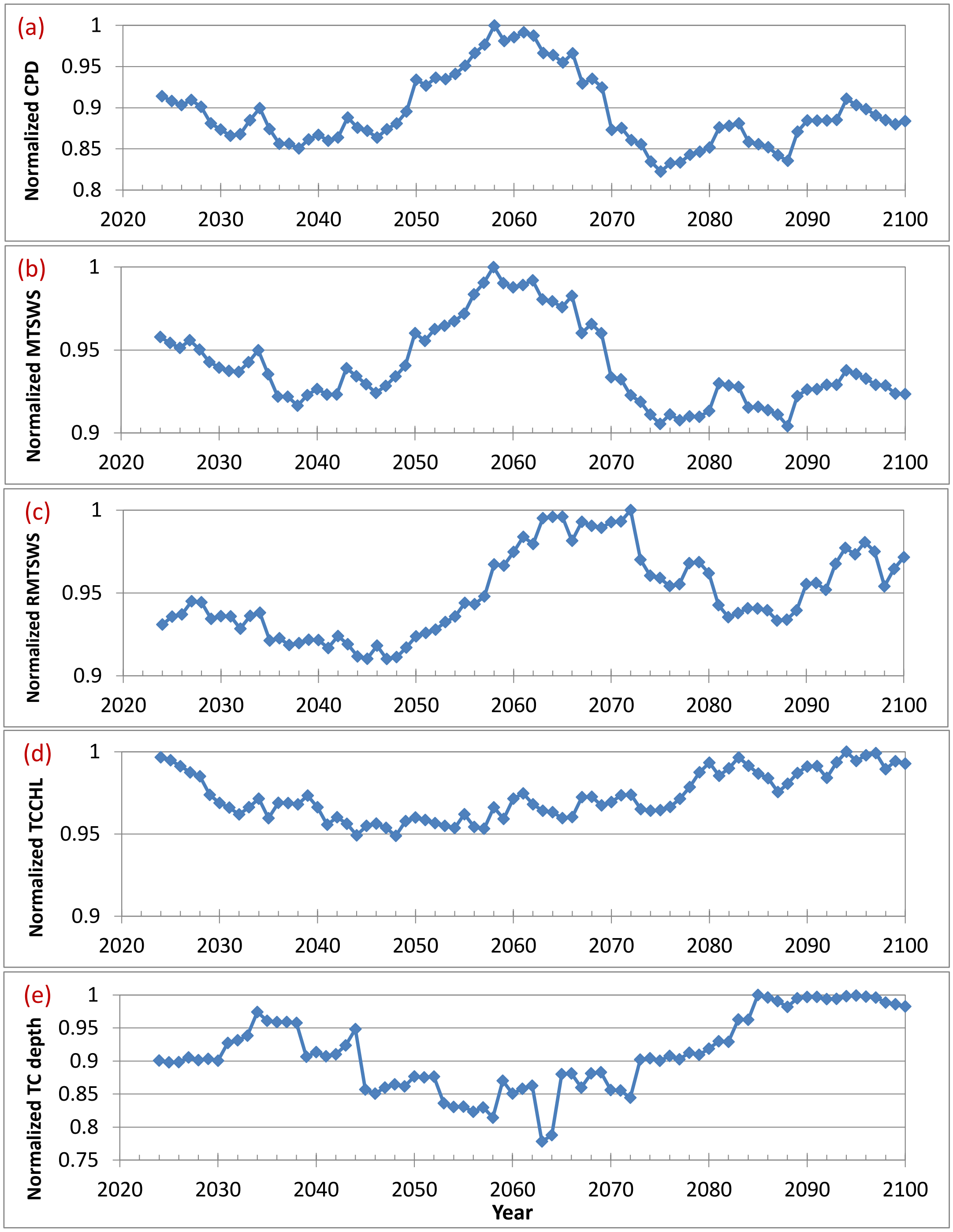


Figure 1: Evolution of the moving average of normalized TC properties during the 21st century. An averaging window of 20 years was used so that the first value is given for 2024 (the first year of the climate projection being 2005). Averaged TC properties were normalized by dividing each dimensional series by the maximum value in the series given in Table 1. As a result, dimensional TC properties can be recovered by multiplying the series by the corresponding maximum given in this table. (a) gives the normalized CPD. (b) gives the normalized MTSWS. (c) gives the normalized RMTSWS. (d) gives the normalized TCCHL. Finally (e) gives the normalized TC depth.

studies (e.g. Bender et al., 2010; Knutson et al., 2013) generally predict an overall decrease in TC frequency and an increase in the frequency of the most intense TCs by the end of the 21$^{st}$ century, whereas in our case, such rarefaction and intensification only occurs during the middle of the 21$^{st}$ century.

Besides, Figure 1c shows that the RMTSWS tends to be slightly smaller compared to 2005-2024 during the first half of the century, whereas it is significantly larger at the beginning of the second half of the century (from approximately 2060 to 2080 in Figure 1c), and only slightly larger at the end of the century. Interestingly, the variations of the RMTSWS do not match the variations of the TCCHL. In fact, the TCCHL does not significantly vary during the 21$^{st}$ century, although it is slightly smaller during most of the century (from 2025 to 2080 in Figure 1d) compared to 2005-2024. Finally, Figure 1e shows that the TC depth tends to be slightly larger at the beginning of the 21$^{st}$ century (from 2030 to 2045 in Figure 1e) compared to 2005-2024, whereas it is smaller in the middle of the century (from 2045 to approximately 2075), and increases significantly at the end of the century.

## References

Bender, M. A. et al. (2010). "Modeled Impact of Anthropogenic Warming on the Frequency of Intense Atlantic Hurricanes". In: *Science* 327.5964, pp. 454–458. ISSN: 0036-8075. DOI: 10.1126/science.1180568.

Knutson, T. R. et al. (2013). "Dynamical Downscaling Projections of Twenty-First-Century Atlantic Hurricane Activity: CMIP3 and CMIP5 Model-Based Scenarios". In: *Journal of Climate* 26.17, pp. 6591–6617. DOI: 10.1175/JCLI-D-12-00539.1.
