# Supplementary material for: Investigation of Intense Precipitation from Tropical Cyclones during the 21st Century by Dynamical Downscaling of CCSM4 RCP 4.5
Source: Int J Environ Res Public Health. 2019 Feb 26;16(5):687. doi: 10.3390/ijerph16050687 (PMC6427206; doi:10.3390/ijerph16050687)
Supplement: Supplementary file 1 [file ijerph-16-00687-s001.zip › supplementary_files/Suppl.Mat._Validation_Detection&Tracking.pdf]

This document supplements Section 3 of the article regarding the validation of the detection and tracking algorithms. More specifically, Figure 1 presents the results for the reconstruction of the tracks of Category 4 and 5 hurricanes from 2004 to 2017 in the Climate Forecast System Reanalysis (CFSR) using the detection and tracking algorithms.

It is observed that these tracks are overall reconstructed satisfactorily. Yet, in most cases, the programs did not detect the early part of the track for which a tropical cyclone is of tropical storm strength. Furthermore, performance was poorer in the region around the Caribbean Islands than over the open ocean, which is certainly due to the effects of the islands (i.e. transition land/ocean as well as topography effects) on the different atmospheric fields. For instance, the programs failed to predict the transition of Hurricane Wilma (2005) from the Gulf of Mexico to the North Atlantic Ocean.

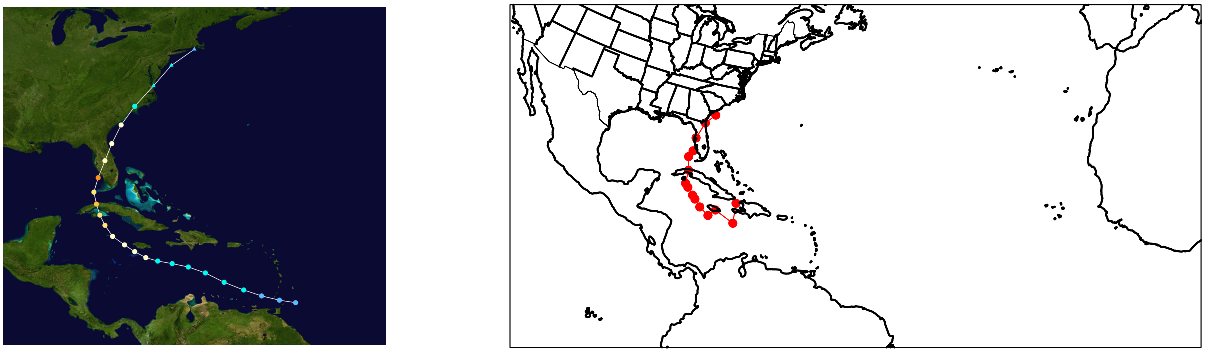

Hurricane Charley (2004, Category 4)

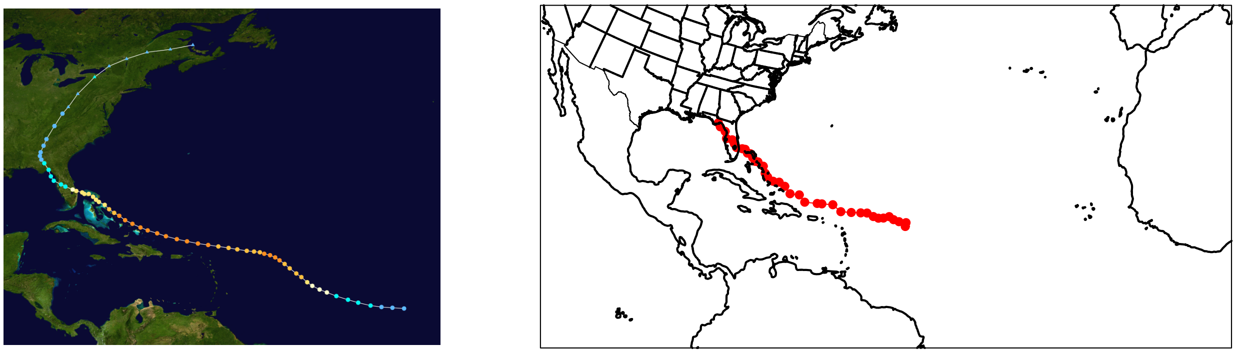

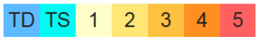

Hurricane Frances (2004, Category 4)

Figure 1: The right column shows the tracks until the time of landfall (if any) of Category 4 and 5 Atlantic hurricanes from 2004 to 2017 obtained by applying the detection and tracking algorithms presented in Section 3 of the article to CFSR. Red dots give the TC location every 6 hours. The left column shows the observed tracks obtained from the free online encyclopedia *Wikipedia*. The color of the dots along the track gives the TC intensity based on the Saffir-Simpson Hurricane Wind Scale. *TD* stands for *tropical depression*, *TS* stands for *tropical storm*, and the numbers correspond to hurricane category from 1 to 5.

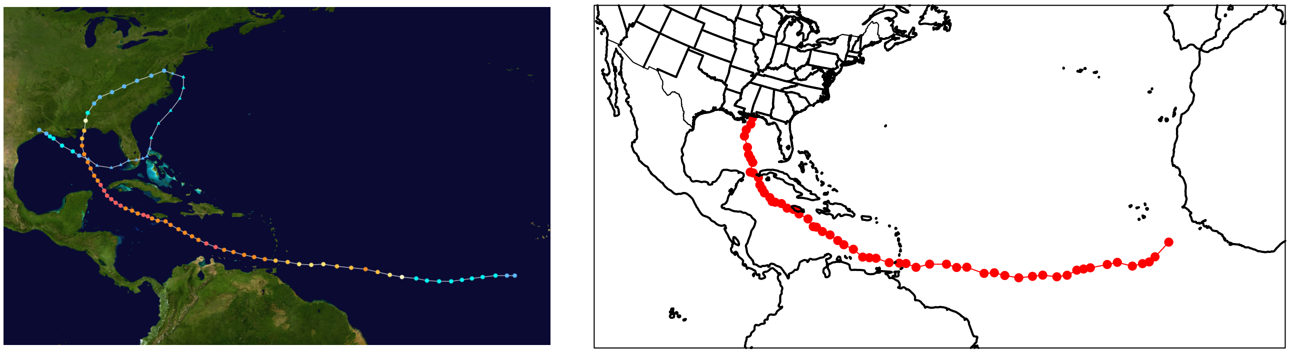

Hurricane Ivan (2004, Category 5)

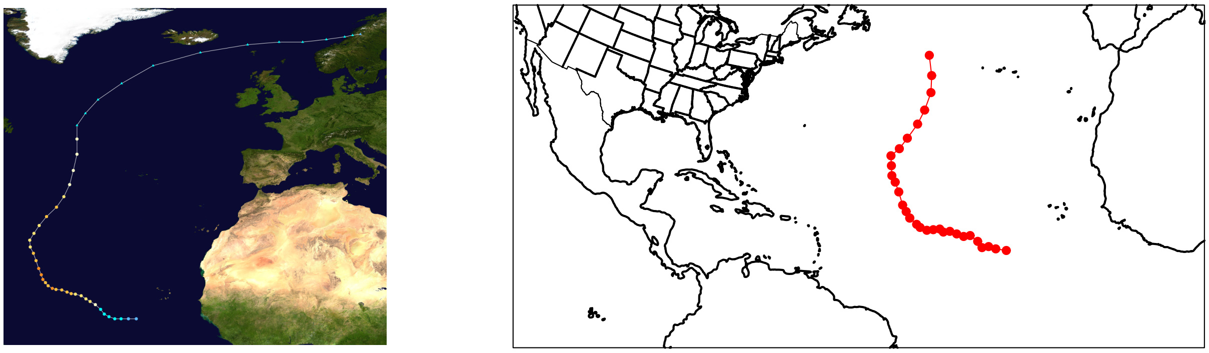

Hurricane Karl (2004, Category 4)

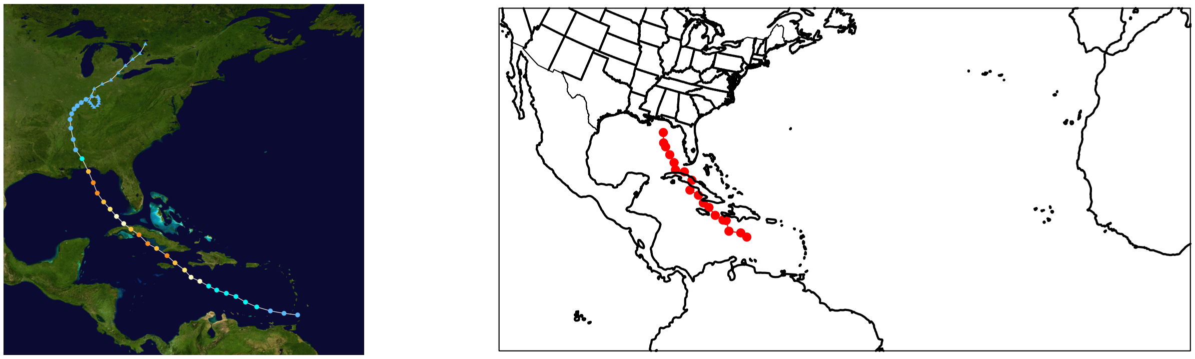

Hurricane Dennis (2005, Category 4)

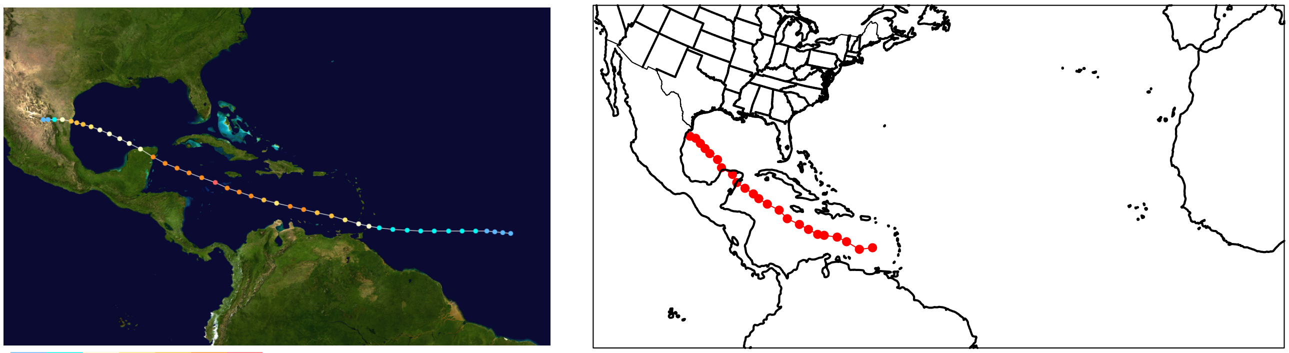

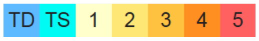

Hurricane Emily (2005, Category 5)

Figure 1 (Cont.)

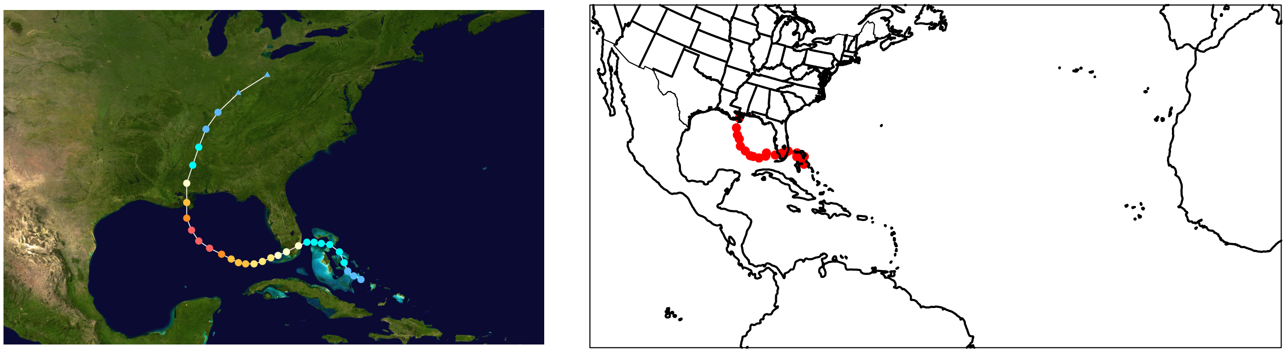

Hurricane Katrina (2005, Category 5)

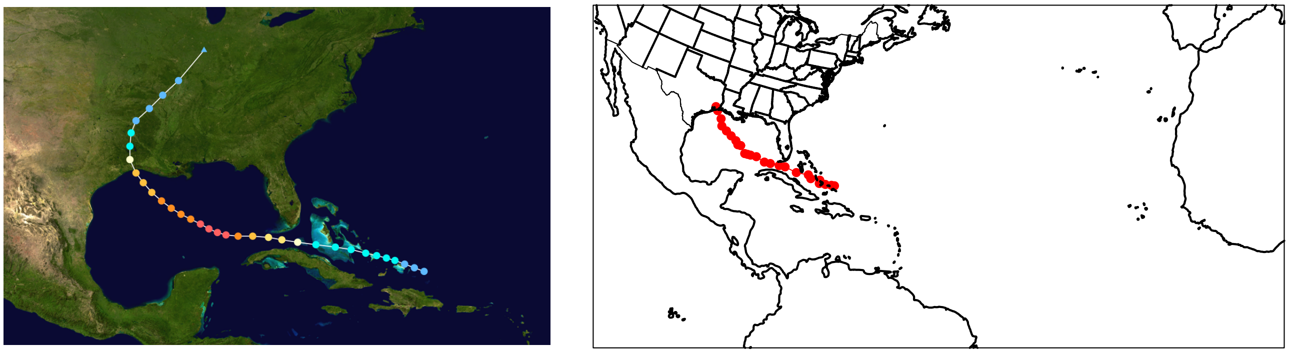

Hurricane Rita (2005, Category 5)

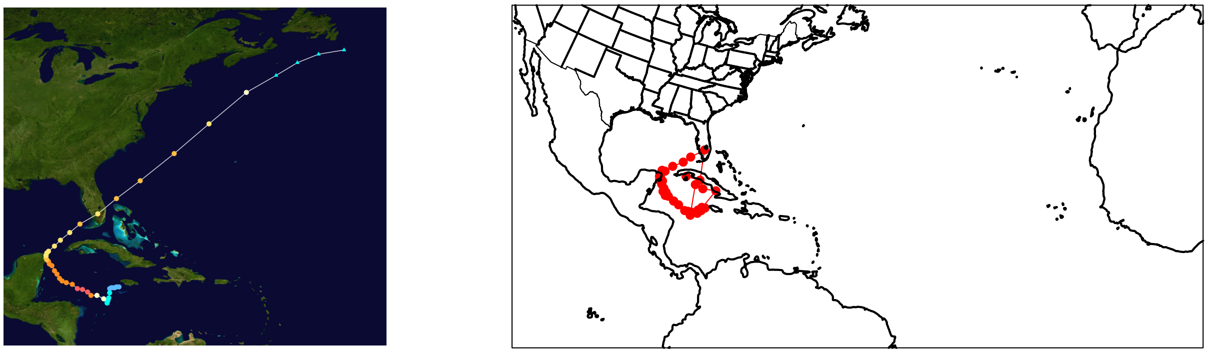

Hurricane Wilma (2005, Category 5)

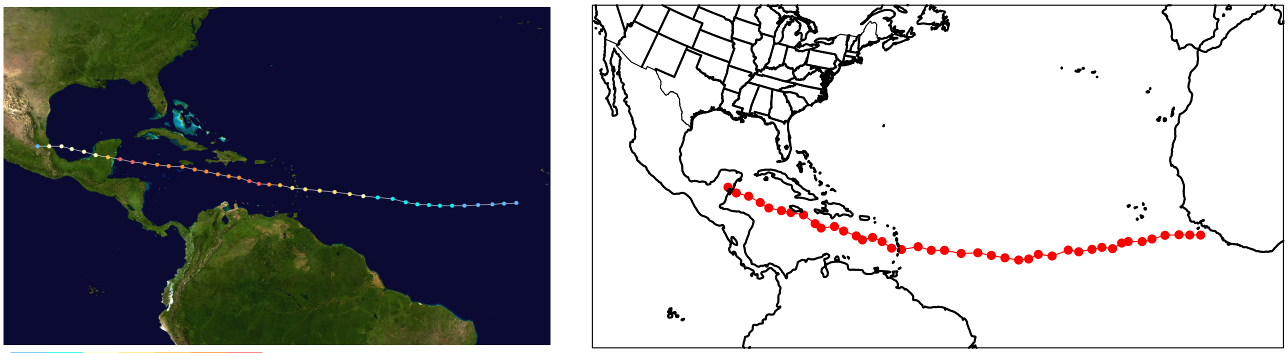

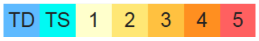

Hurricane Dean (2007, Category 5)

Figure 1 (Cont.)

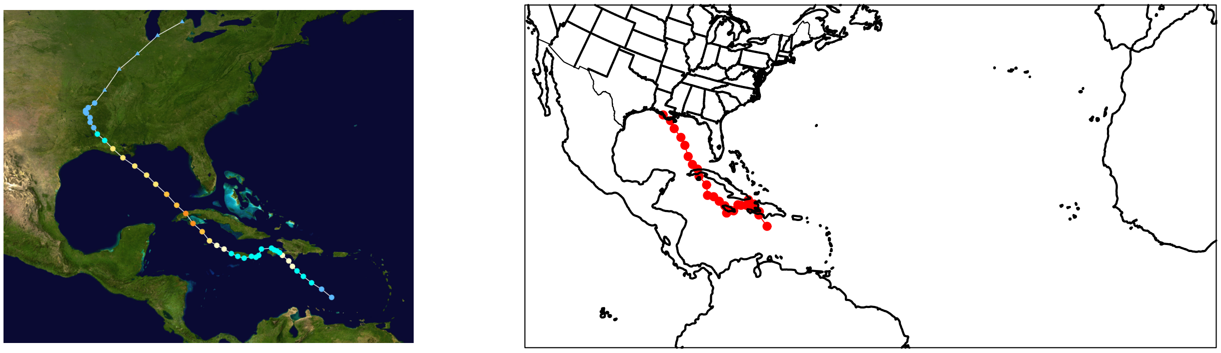

Hurricane Gustav (2008, Category 4)

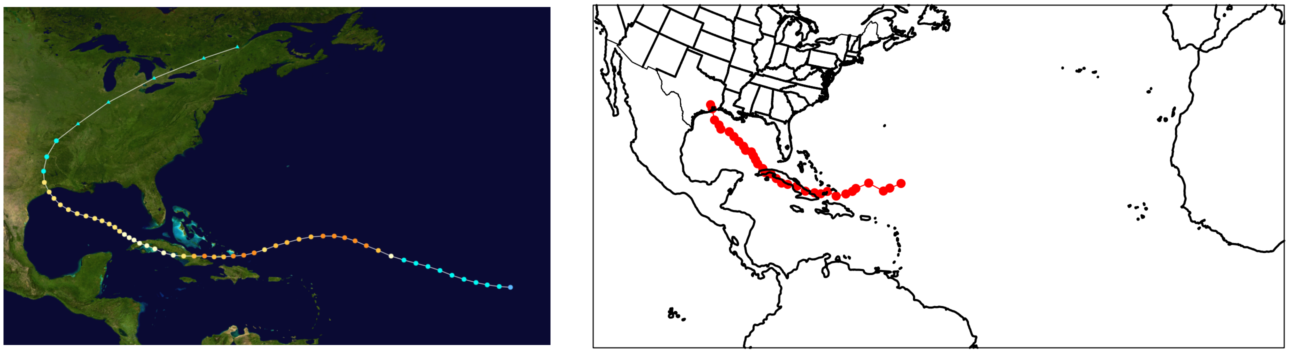

Hurricane Ike (2008, Category 4)

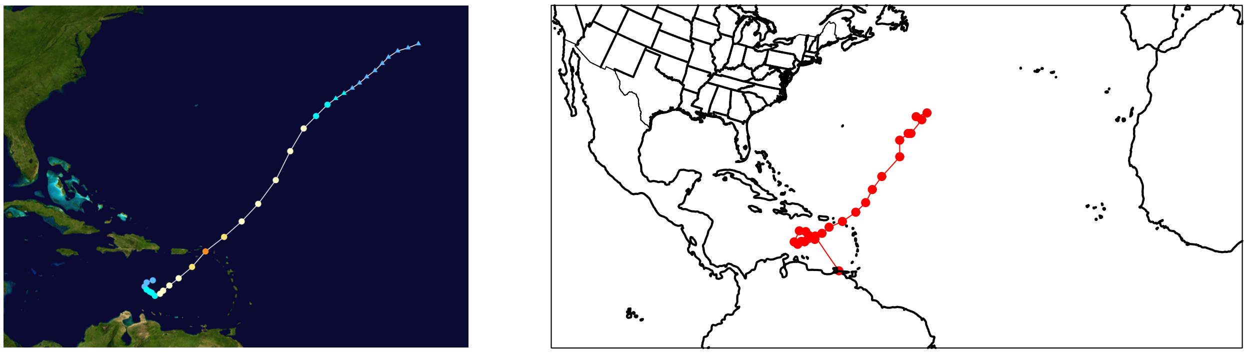

Hurricane Omar (2008, Category 4)

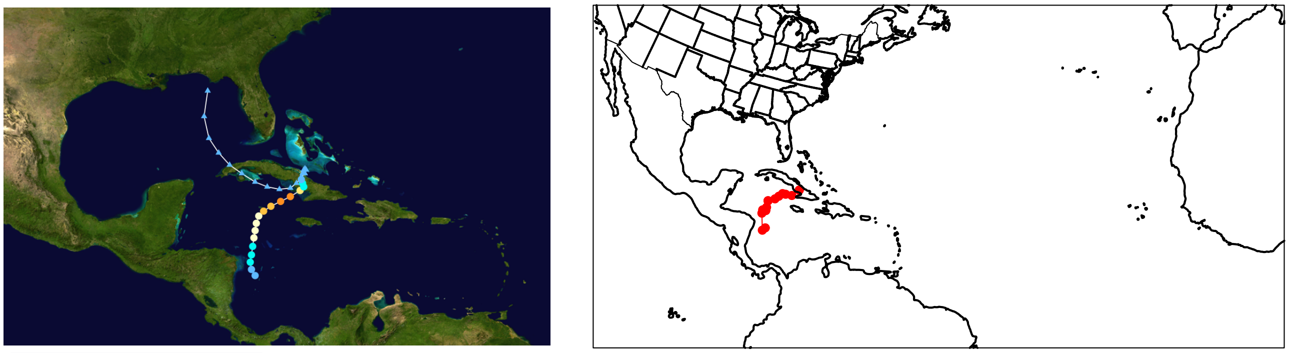

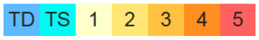

Hurricane Paloma (2008, Category 4)

Figure 1 (Cont.)

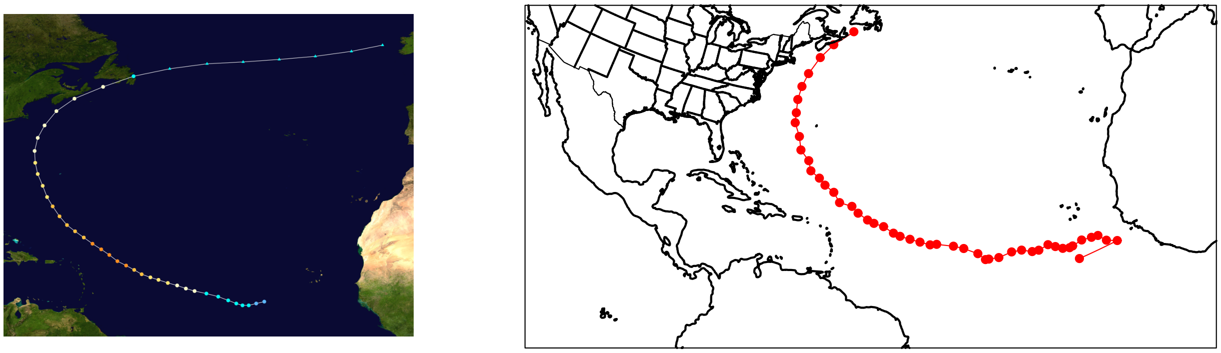

Hurricane Bill (2009, Category 4)

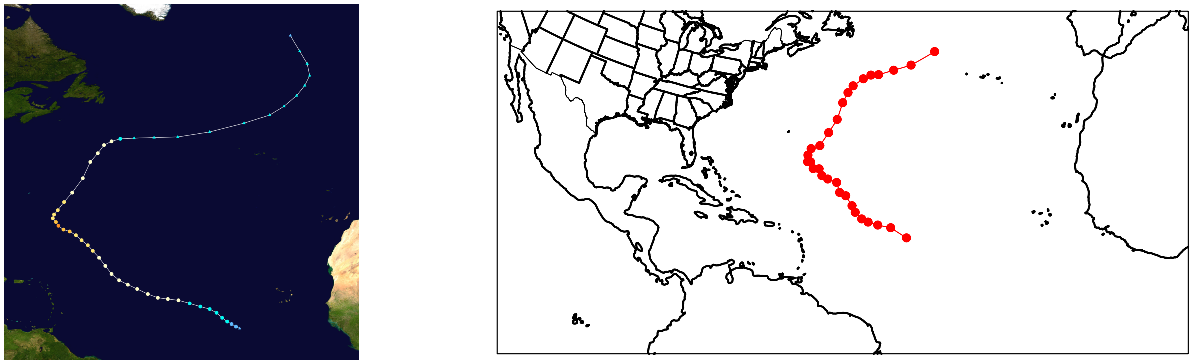

Hurricane Danielle (2010, Category 4)

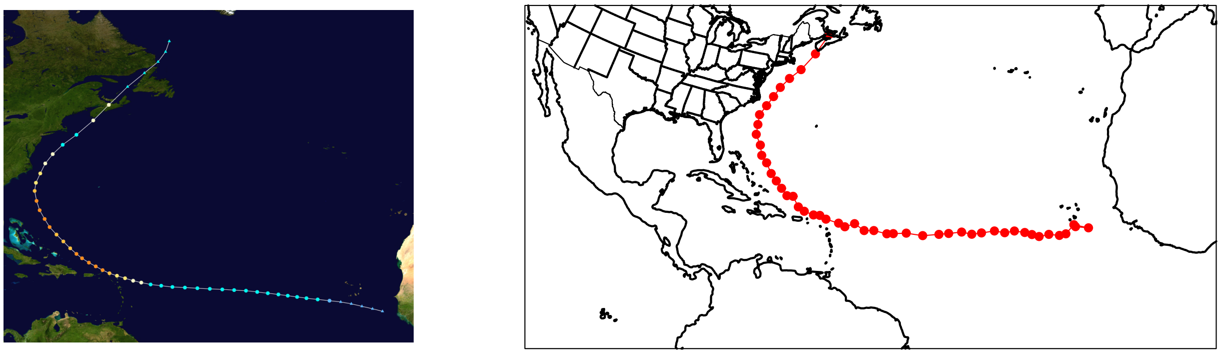

Hurricane Earl (2010, Category 4)

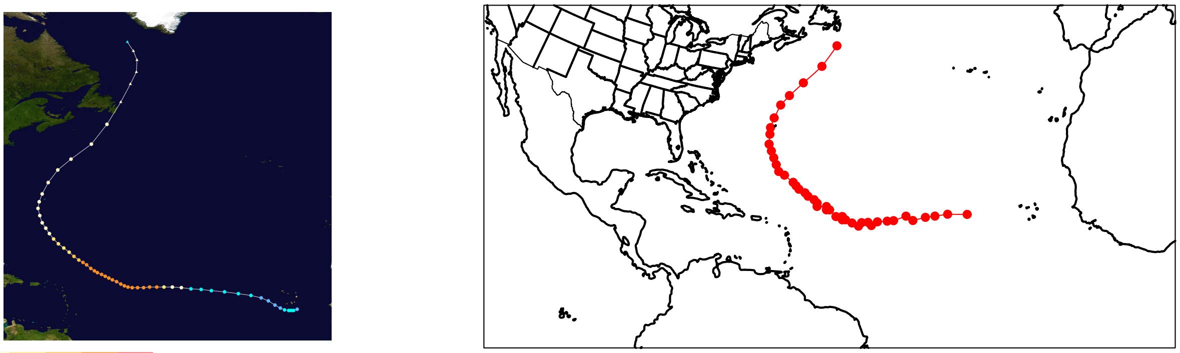

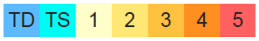

Hurricane Igor (2010, Category 4)

Figure 1 (Cont.)

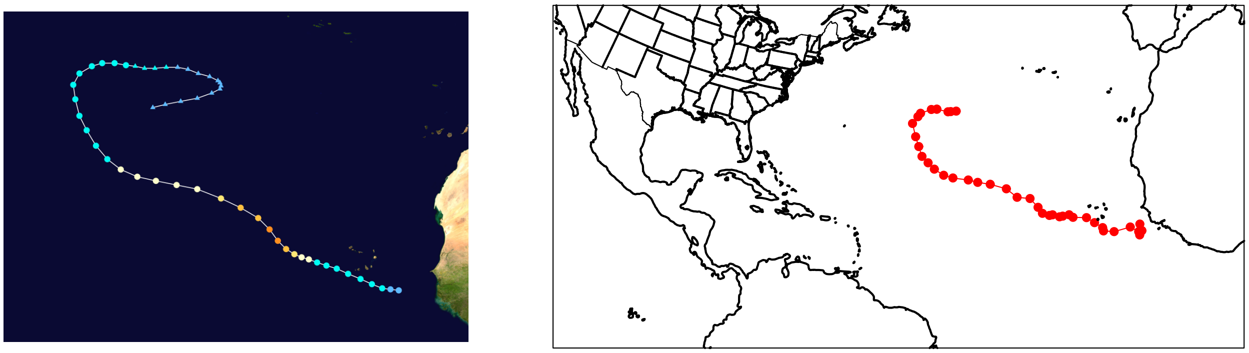

Hurricane Julia (2010, Category 4)

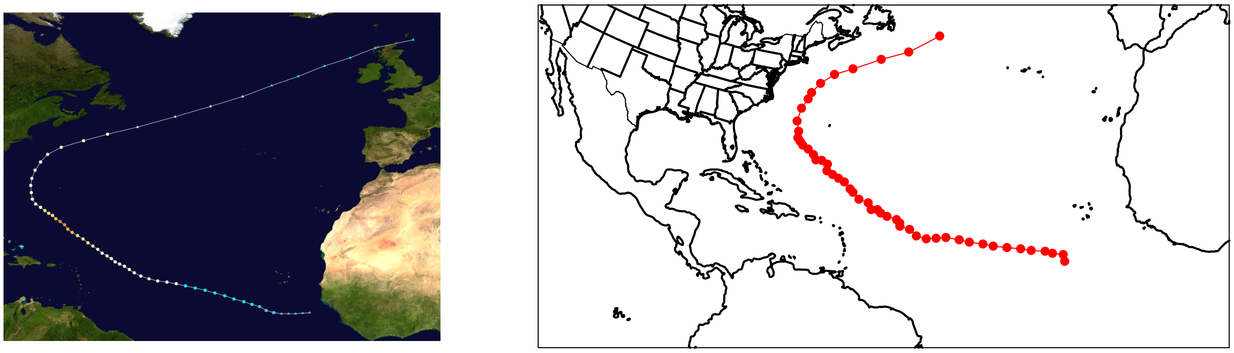

Hurricane Katia (2011, Category 4)

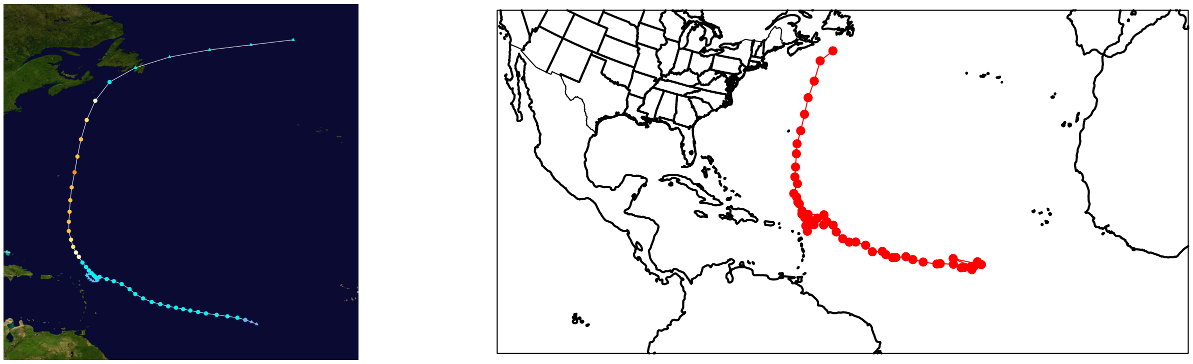

Hurricane Ophelia (2011, Category 4)

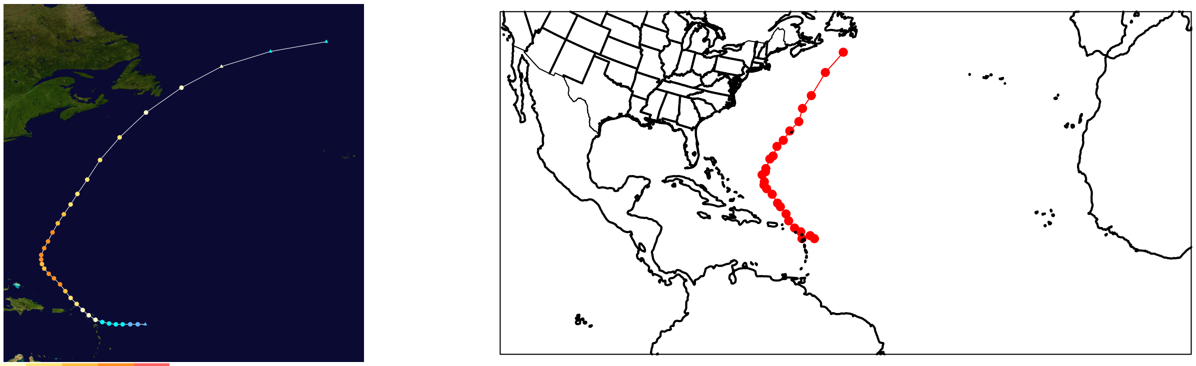

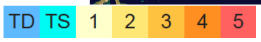

Hurricane Gonzalo (2014, Category 4)

Figure 1 (Cont.)

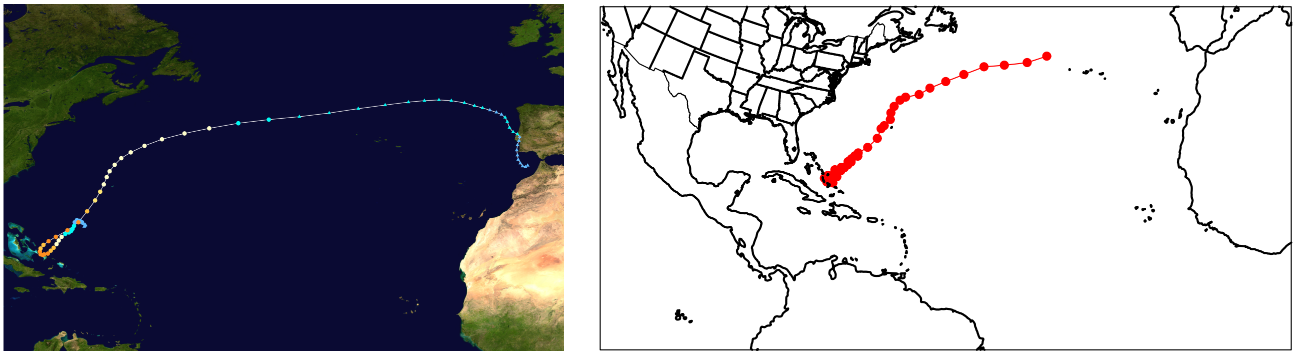

Hurricane Joaquin (2015, Category 4)

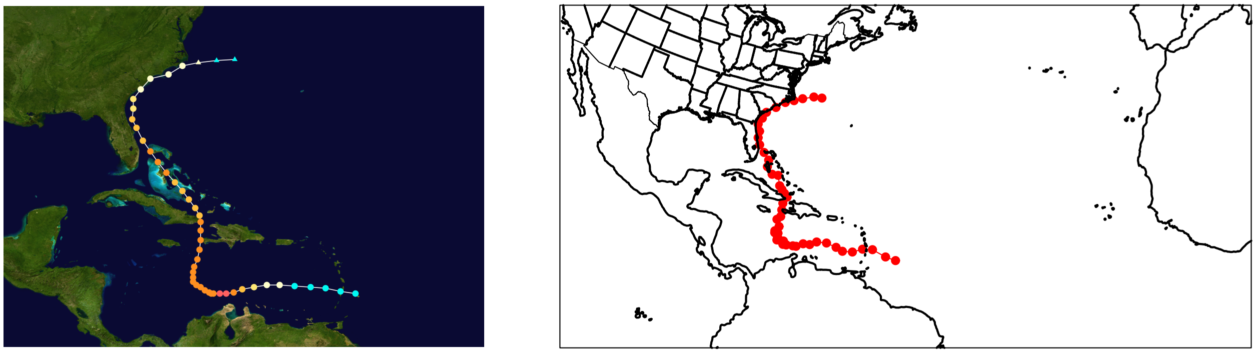

Hurricane Matthew (2016, Category 5)

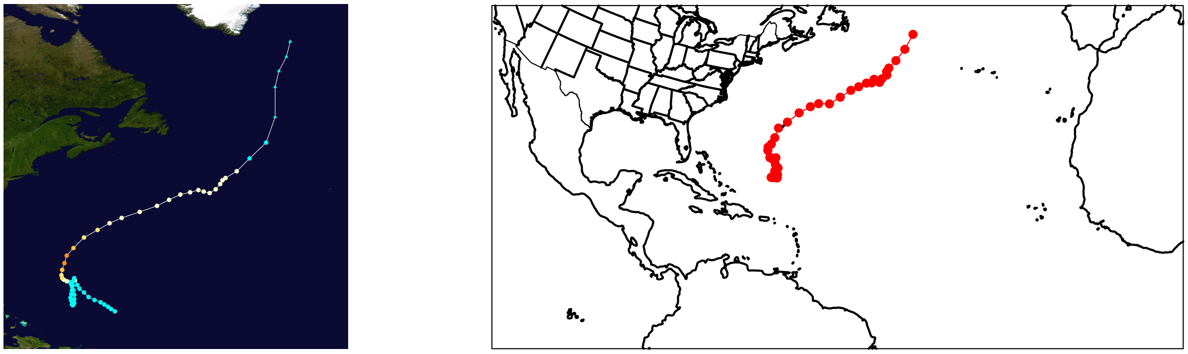

Hurricane Nicole (2016, Category 4)

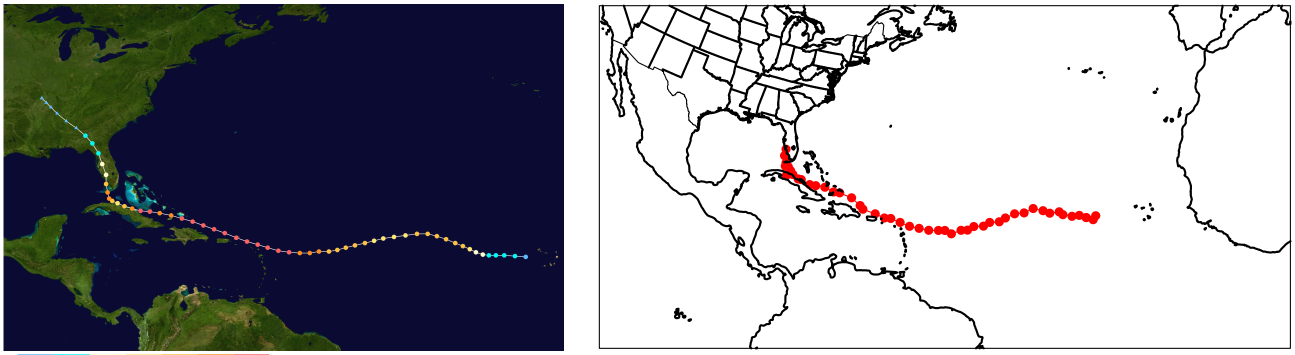

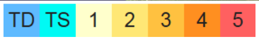

Hurricane Irma (2017, Category 5)

Figure 1 (Cont.)

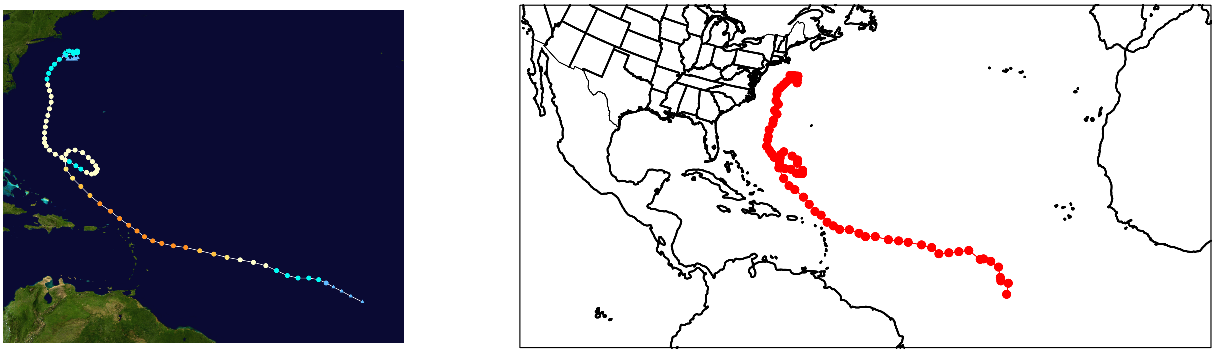

Hurricane Jose (2017, Category 4)

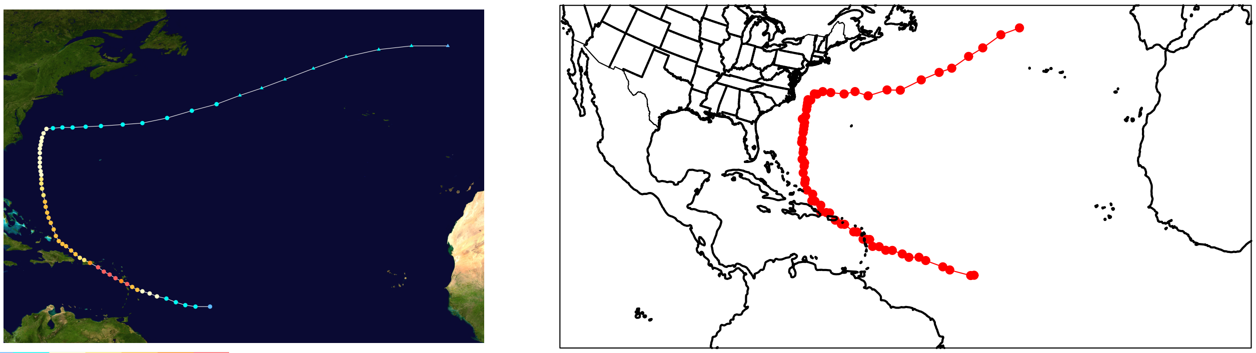

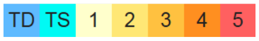

Hurricane Maria (2017, Category 4)

Figure 1 (Cont.)
